# Virome Diversity and Host-Associated Heterogeneity in Mosquitoes and Biting Midges Across Yunnan, China

**DOI:** 10.3390/v18101109

**Published:** 2026-10-08

**Authors:** Honglin Su, Kai Zhao, Yuqiong Li, Zongti Shao, Xinglou Yang, Jian Wang, Shengyao Chen

**Affiliations:** 1 Key Laboratory of Genetic Evolution & Animal Models, Yunnan International Joint Laboratory of Zoonotic Viruses, Yunnan Key Laboratory of Biodiversity Information, Kunming Institute of Zoology, Chinese Academy of Sciences, Kunming 650201, China; suhonglin@mail.kiz.ac.cn (H.S.); zhaokai@mail.kiz.ac.cn (K.Z.); yangxinglou@mail.kiz.ac.cn (X.Y.); 2 Kunming College of Life Science, University of Chinese Academy of Sciences, Kunming 650204, China; 3 Yunnan Institute of Endemic Diseases Control and Prevention, Dali 671000, China; 13577265787@163.com (Y.L.); shaozt_923@163.com (Z.S.)

**Keywords:** mosquitoes, biting midges, virome, host association, Yunnan, Jingmenvirus

## Abstract

Mosquitoes and biting midges harbor diverse viruses, but the factors shaping their viromes remain incompletely understood. We analyzed 35 pooled libraries from seven host groups across 15 locations in Yunnan, China, sampled in June 2025, using total-RNA sequencing, viral annotation, phylogenetics, and community analyses. We retained 1295 viral contigs grouped into 221 annotation-defined viral taxonomic units (VTUs) spanning 24 families; 107 VTUs were supported only by protein homology. Banna virus-related sequences, an *Aedes*-associated flavivirus, and four putative components of a divergent Jingmenvirus-related candidate were identified. Host group was associated with family-level virome composition after adjustment for location and sequencing depth (R^2^ = 0.213, *p* = 0.0052), and the association persisted across alternative feature definitions. *Culex tritaeniorhynchus* showed the greatest cumulative VTU richness, although its ranking changed when library number was equalized. Most VTUs detected in this host occurred at one sampled site, without a significant monotonic distance-decay pattern. This study expands knowledge of mosquito-associated and biting midge-associated viral diversity in Yunnan, identifies representative lineages for further characterization, and documents host-associated differences in the detected viromes.

## 1. Introduction

Hematophagous arthropods transmit viruses of major public-health and veterinary importance [1,2]. Specifically, within the order Diptera, mosquitoes and biting midges represent vectors of such viruses. Their viromes comprise arboviruses and insect-associated viruses, and pooled sequencing can recover viral sequences derived from blood meals, parasites, microorganisms, plants, or environmental material [3,4,5]. Metagenomic and metatranscriptomic detections must therefore be interpreted with reference to pathogen surveillance and the broader viral diversity associated with these hosts.

Viral communities associated with mosquitoes and biting midges vary between host taxa and locations. Studies at different spatial and temporal scales have repeatedly identified the host’s species or phylogeny as major correlates of virome composition. Geography, season, habitat, and local environmental conditions influence the occurrence and abundance of particular viruses [6,7,8,9,10]. Regions with high vector diversity provide opportunities to characterize viromes across diverse host groups and locations.

Yunnan Province in southwestern China shares borders with Myanmar, Laos, and Vietnam. Its terrain spans lowlands, subtropical montane forests, and high-elevation plateau ecosystems along steep climatic and elevational gradients [11,12]. This heterogeneity supports exceptional biological diversity, including nearly 300 recorded *Culicidae* species [13]. Dengue-vector *Aedes* species occur mainly in southern Yunnan, in areas including Xishuangbanna, Dehong, Honghe, Wenshan, and Pu’er [14]. Surveys in the Lancang River basin have associated *Culicidae* richness with latitude, elevation, temperature, and precipitation [15]. Earlier surveys have reported *Cx. tritaeniorhynchus* as dominant in several areas and *Anopheles sinensis* as abundant in some habitats [16,17]. Through regional surveillance, Japanese encephalitis virus, dengue virus, Banna virus, and numerous viruses associated with *Culicidae* have been detected [18,19,20,21]. Metatranscriptomic studies have also reported a pronounced seasonal and host-associated structure in local *Culicidae* viromes [7,19]. Most regional studies, however, have targeted individual pathogens, border localities, animal farms, or restricted sampling periods [7,18,19,20,21]. The distribution of viral diversity among host groups and locations across the province remains less well characterized.

Separating these associations in field collections is difficult because climate and habitat influence host-group occurrence while sampling effort and sequencing depth are often uneven [6,7,8,9,10,22,23]. Analytical resolution adds a further complication. Family-level profiles can conceal turnover among related viruses, whereas contig-level comparisons can inflate diversity by counting fragments or genome segments separately. Nucleotide similarity provides relatively strong support for known-virus assignments. Protein-level homology is more sensitive to divergent sequences but often permits less precise taxonomic placement. A viral sequence detected in a pooled library also cannot be assigned to an individual insect.

In this study, we characterized the viral sequences detected in pooled mosquitoes and biting midges from Yunnan. We assessed how richness and composition varied between host groups, locations, developmental stages, and broad climate zones. We also examined the phylogenetic relationships of representative sequences and tested whether host-group associations remained stable after accounting for sampling design.

## 2. Materials and Methods

### 2.1. Sample Collection and Host Identification

In a cross-sectional collection in June 2025, 35 pooled libraries representing seven host groups and 15 locations in Yunnan were selected for sequencing. Adult mosquitoes and biting midges were collected using photocatalytic mosquito traps (LTS-M02, Wuhan Jixing Environmental Technology Co., Ltd., Wuhan, China). *Culex* spp. larvae were collected from sewer habitats with a hand net and then immobilized under cold conditions and stored in cryogenic tubes until processing. Library composition and collection-site metadata for all 35 libraries are provided in Table S1.

Specimens were identified morphologically using standard taxonomic criteria and assigned to the finest host or developmental category consistently available at each location. The sequenced pools comprised *Cx. tritaeniorhynchus*, *Culex quinquefasciatus* (*Culex pipiens quinquefasciatus*), *Anopheles sinensis*, *Aedes albopictus*, *Armigeres subalbatus*, *Ceratopogonidae*, and *Culex* spp. larvae. Host-group associations therefore refer to pooled taxonomic or developmental groups rather than to individual insects, and taxonomic resolution differed between groups. Table S1 lists the library identifier, host group, locality, administrative region, coordinates, climate-zone category, and available specimen information for each library. Before pooling, specimens were sorted by collection site, morphological host category, and collection batch.

### 2.2. RNA Extraction and Sequencing

Up to 50 specimens were randomly selected from the same site, host group, and collection batch for each pooled library; when fewer than 50 were available, all were used. Each pool was homogenized under cold conditions, and total RNA was extracted with the Virus DNA/RNA Extraction Kit 2.0 (RM01-04, Vazyme, Nanjing, China) according to the manufacturer’s instructions. Libraries were prepared from total RNA without rRNA depletion or poly(A) enrichment; the libraries were not strand-specific. Sequencing was performed on an Illumina NovaSeq 6000 platform to generate 150 bp paired-end reads.

### 2.3. Read Filtering, Host and rRNA Depletion, and De Novo Assembly

Raw reads were quality-filtered with fastp [24]. The resulting clean reads were retained for read remapping and the library-depth denominator. A depletion database was assembled from available NCBI genomes for the represented host taxa and from curated ribosomal RNA sequences. Clean reads were aligned to this database using Bowtie2 v2.5.4 [25], and reads mapping to host genomes or rRNA were removed. The remaining unmapped reads were assembled de novo with MEGAHIT v1.2.9 [26]. Contigs shorter than 500 bp were discarded.

### 2.4. Viral Contig Discovery, Annotation, and Evidence Classes

Candidate viral contigs were identified by complementary protein and nucleotide level searches. DIAMOND BLASTx [27] searches used the NCBI non-redundant protein database (nr; dated 9 April 2026; 1,047,403,710 sequences and 397,849,455,130 residues), followed by screening against a curated RNA-dependent RNA polymerase (RdRp) database. BLASTn searches used the NCBI nucleotide database (nt; dated 13 April 2026; 124,491,017 sequences and 3,677,374,362,840 bases). Both local databases used BLASTDB version 5. Contigs of at least 500 bp supported by at least eight remapped reads were retained; a comparable study of pooled mosquito viromes applied a minimum of 10 mapped reads [10]. Read remapping was used to check consistency between assembled contigs and their underlying reads [28]. Candidates retained solely through protein searches required a viral hit with an E-value ≤ 1 × 10^−4^ and an alignment of at least 40 amino acids. They also required amino-acid identity of approximately 20% or greater and an open reading frame, conserved motif, or domain organization consistent with the annotation. Nucleotide-supported assignments required at least 70% nucleotide identity to a viral reference; candidates whose top-scoring BLASTn match was non-viral were excluded. For partial genes, alignment length, contig length, and protein architecture were evaluated jointly.

Contigs with a qualifying viral nucleotide match were assigned to the nucleotide-supported class. All other retained candidates identified by searches of nr or the curated RdRp database were assigned to the protein-supported class, including candidates recognized through conserved polymerase domains. Contigs with retained viral evidence but no stable family-level placement were classified as family-unresolved [29]. Sensitivity analyses tested whether host-group associations persisted among nucleotide-supported VTUs, among detections with at least 20 reads, and among VTUs present in at least two libraries.

### 2.5. Definition of VTUs, Abundance Matrices, and Phylogenetic Analysis

Quality-filtered reads before host and rRNA depletion were mapped back to the retained viral contigs with Bowtie2 [25] and summarized with SAMtools 1.24. Contig RPM was calculated as mapped reads divided by total pre-depletion clean reads in the same library, multiplied by one million. The same total-read count was used as the sequencing-depth covariate. Each retained contig carried a curated reference-based species or species-like label in the BN_Species field. Contigs sharing the same label were grouped into a VTU, following an approach developed for shotgun virome contigs that may represent non-overlapping regions of the same viral genome [10]. RPM values were summed within each VTU and library, yielding 221 VTUs; 136 contained multiple contigs and 85 were represented by a single contig (median, 2 contigs per VTU; range, 1–59). VTUs served as reference-associated analytical groups, not sequence-delimited species, strains, or complete genomes. Grouping by annotation, without a pairwise identity threshold, reduced repeated counting of fragments or segments assigned to one reference and retained divergent sequences detected by protein homology. Sparse references can merge distinct viruses under one label or split fragments of one virus between labels. Stable identifiers VTU001-VTU221 are listed in Table S2, and the complete 1295-contig mapping is provided in the Contig_to_VTU sheet. Family-level matrices retained the contig-level assignments, including the family-unresolved category.

Assembled viral proteins and reference homologs were aligned with MAFFT v7 using the --localpair and --maxiterate 1000 options [30]. Existing family-level alignments were filtered with trimAl v1.4 to remove gap-rich and poorly aligned sites [31]. For the *Orthomyxoviridae* PB1 and *Parvoviridae* NS1 alignments, columns occupied in fewer than 50% of sequences were removed, leaving 785 and 591 amino-acid positions, respectively. The final *Peribunyaviridae*, *Phenuiviridae*, and *Rhabdoviridae* alignments contained 1841, 538, and 819 positions. The focused Banna VP1 analysis, including LF_M39 (OR476072), retained 1115 positions; the WC-15 NS5/RdRp and NS3 alignments contained 584 and 596 positions (Tables S14 and S15).

Maximum-likelihood trees were inferred with IQ-TREE v2 [32]. Substitution models for the family-level and focused Banna virus analyses were selected with ModelFinder [33]. The *Peribunyaviridae* and *Rhabdoviridae* trees were re-estimated under their selected models after restriction to the 35-library analysis set. Branch support was estimated from 1000 ultrafast bootstrap and 1000 SH-aLRT replicates [34]; the WC-15 trees used the same support scheme under the selected LG+I+G4 model. The reversible substitution models yielded unrooted trees. Display orientation followed the saved tree files. The Banna virus analysis specified OUT_1 and the *Aedes*-associated flavivirus analysis specified OUT_1 and OUT_2 as outgroups. Other panels retained the recorded display orientation without additional rerooting. Panel-specific display and outgroup settings are documented in Table S14c. Alignments, model reports, and Newick files are provided in File S2, with sequence counts and support values in Tables S14 and S15.

Trees were visualized in R with ggtree [35] and standard phylogenetic plotting functions. Reference clades containing at least three sequences assigned to the same genus were collapsed when the inferred topology supported them. Branches leading to sequences recovered from the Yunnan libraries were colored red, and an aligned strip marked selected genus-level assignments for reference sequences. To keep the overview legible, genus names were shown for well-represented reference groups and for the reference genera nearest to recovered sequences; sparsely represented genera were combined as other reference genera. Recovered sequences were not assigned to a genus solely based on their position in the overview. Figure 5A,B show selected-reference views; full analyses are supplied in File S2. Figure 6A,B show the complete WC-15 trees without reference pruning, also provided in Figure S6.

### 2.6. Ecological and Statistical Analyses

Viral community structure was evaluated at the family and VTU levels. Abundance-based profiles were compared with Bray-Curtis dissimilarity, and binary presence–absence profiles with Jaccard dissimilarity. Host-group associations were tested by PERMANOVA with 9999 permutations. Marginal models accounted for location and log10 total pre-depletion clean reads; additional tests restricted permutations within location. Design checks used multi-host sites and profiles averaged within duplicate host-location cells. PERMDISP tested distances to group spatial medians using 9999 permutations, with small-sample and Lingoes adjustments examined separately. Benjamini-Hochberg correction was applied jointly to seven location- and depth-adjusted VTU sensitivity tests, including abundance and incidence analyses excluding single-contig VTUs.

Within-host spatial turnover, adult-larval differences, and site-level host-virome concordance were evaluated by ordination and permutation tests. Library-level richness was compared between author-defined climate-zone groups by a Wilcoxon rank-sum test. Richness checks used all three-library combinations within host groups, a depth-adjusted negative-binomial model, and rarefaction of retained VTU counts to the lowest pre-depletion clean-read total. Spatial detection patterns were also checked under evidence and read-support filters and after retaining one library per location. Temperature and precipitation information was recorded from weather-application displays during sampling in June 2025, rather than measured directly at the collection sites. Their represented periods and the precipitation definition could not be verified, so they were excluded from quantitative environmental models. Site-level analyses were treated as exploratory because sampling was limited and unbalanced. Full model formulas, preprocessing steps, permutation schemes, duplicate-pool handling, and the terminal-motif procedure are provided in Supplementary Methods S1.

## 3. Results

### 3.1. Sampling Design and Overall Virome Composition

**Figure 1:**
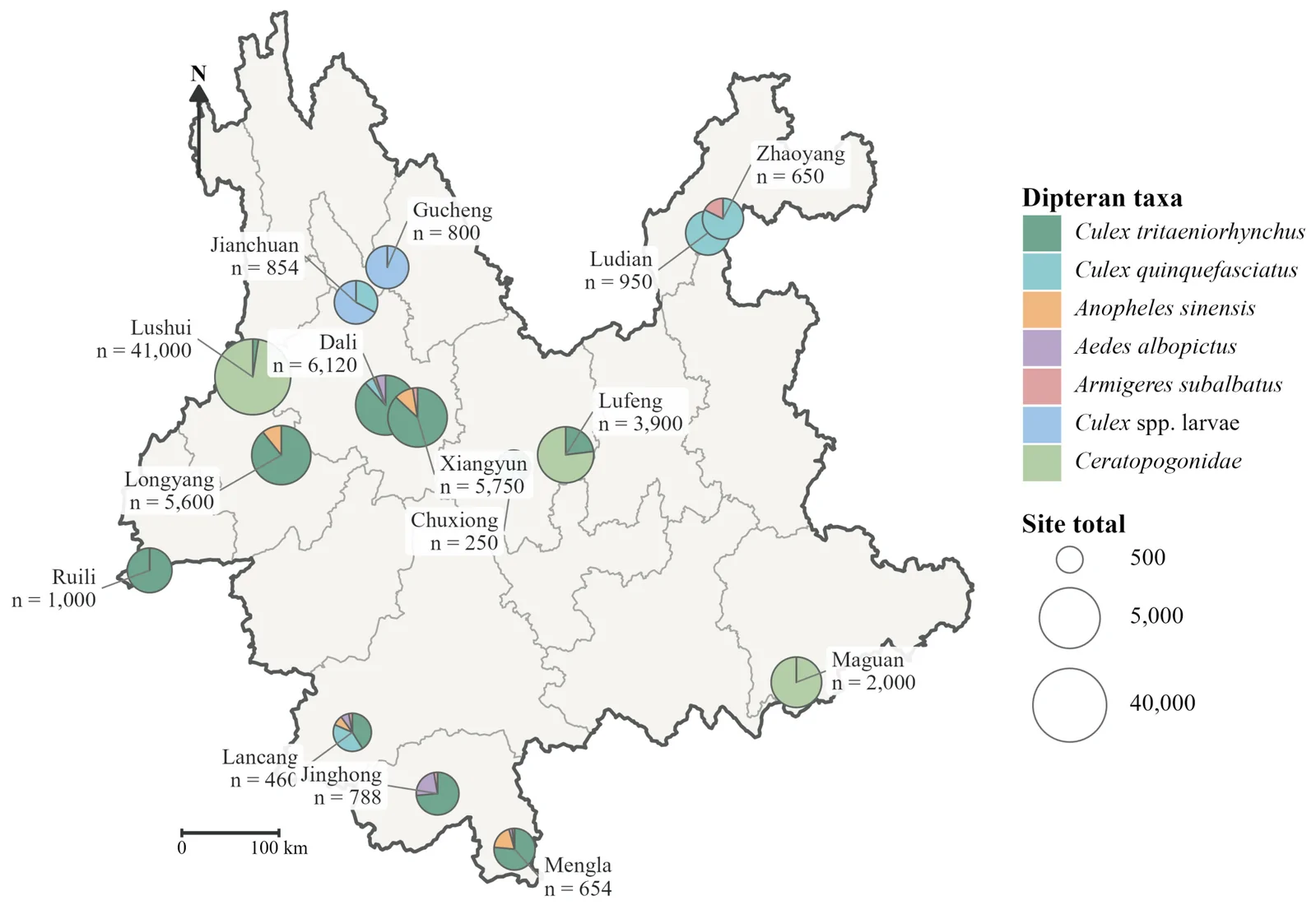
Geographic distribution and taxonomic composition of field-collected *Culicidae* and *Ceratopogonidae* at 15 locations in Yunnan Province, China. Pie segments indicate field host-group composition, and radii are scaled to log10-transformed site totals. Virome analyses used 35 pooled libraries representing 32 sequenced host-group-by-location combinations (Table S1).

The sequencing design comprised 35 pooled libraries from seven host groups in 32 host-group-by-location combinations across 15 locations, including three combinations represented by duplicate libraries (Figure 1; Table S1). Nine locations contained two to five sequenced host groups and contributed 28 libraries; the other six locations contained one host group (Figure S4A; Table S11). All virome composition, richness, and ecological analyses used these 35 libraries only.

**Figure 2:**
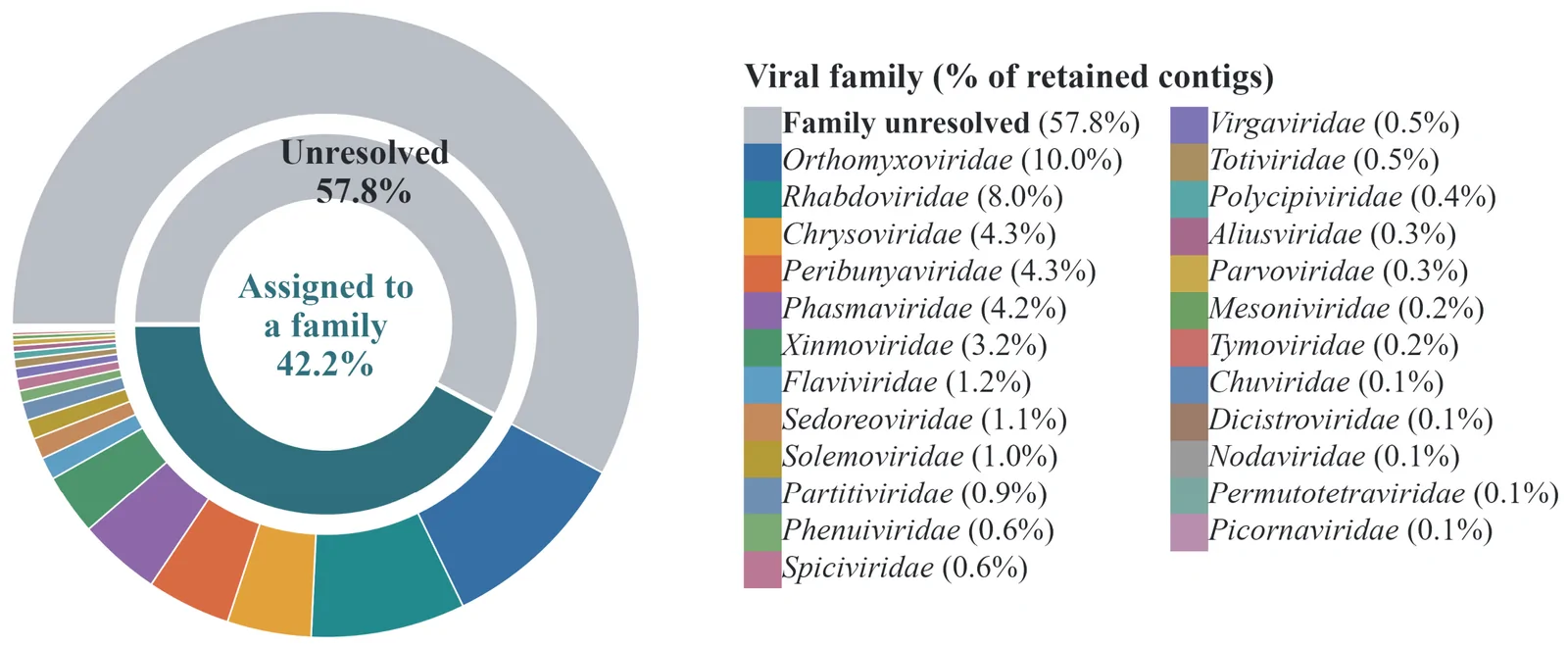
Viral family composition of retained viral contigs. The inner ring contrasts family-assigned and family-unresolved sequences; the outer ring shows the contribution of each viral family. Percentages describe retained contig proportions, not relative viral abundance or complete-genome counts.

The 35 libraries yielded 1295 contigs that met the viral-sequence retention criteria. Contigs sharing the same curated BN_Species label were consolidated into 221 annotation-defined VTUs, 114 of which contained at least one nucleotide-supported contig; the other 107 were supported only by protein-level evidence. Eighty-five VTUs (38.5%) contained one contig, with a median length of 834 nt (IQR, 611–1236 nt; Table S17). Of the retained contigs, 847 (65.4%) were nucleotide-supported and 448 (34.6%) were protein-supported. Assignments spanned 24 viral families plus a family-unresolved category. Among the 749 family-unresolved contigs, 301 (40.2%) matched nucleotide references without an accepted family placement and 448 (59.8%) were retained from nr or RdRp protein matches. The largest assigned fractions belonged to *Orthomyxoviridae* (10.0%), *Rhabdoviridae* (8.0%), *Chrysoviridae* (4.3%), *Peribunyaviridae* (4.3%), and *Phasmaviridae* (4.2%) (Figure 2).

### 3.2. Host-Associated Viral Richness and Family Composition

**Figure 3:**
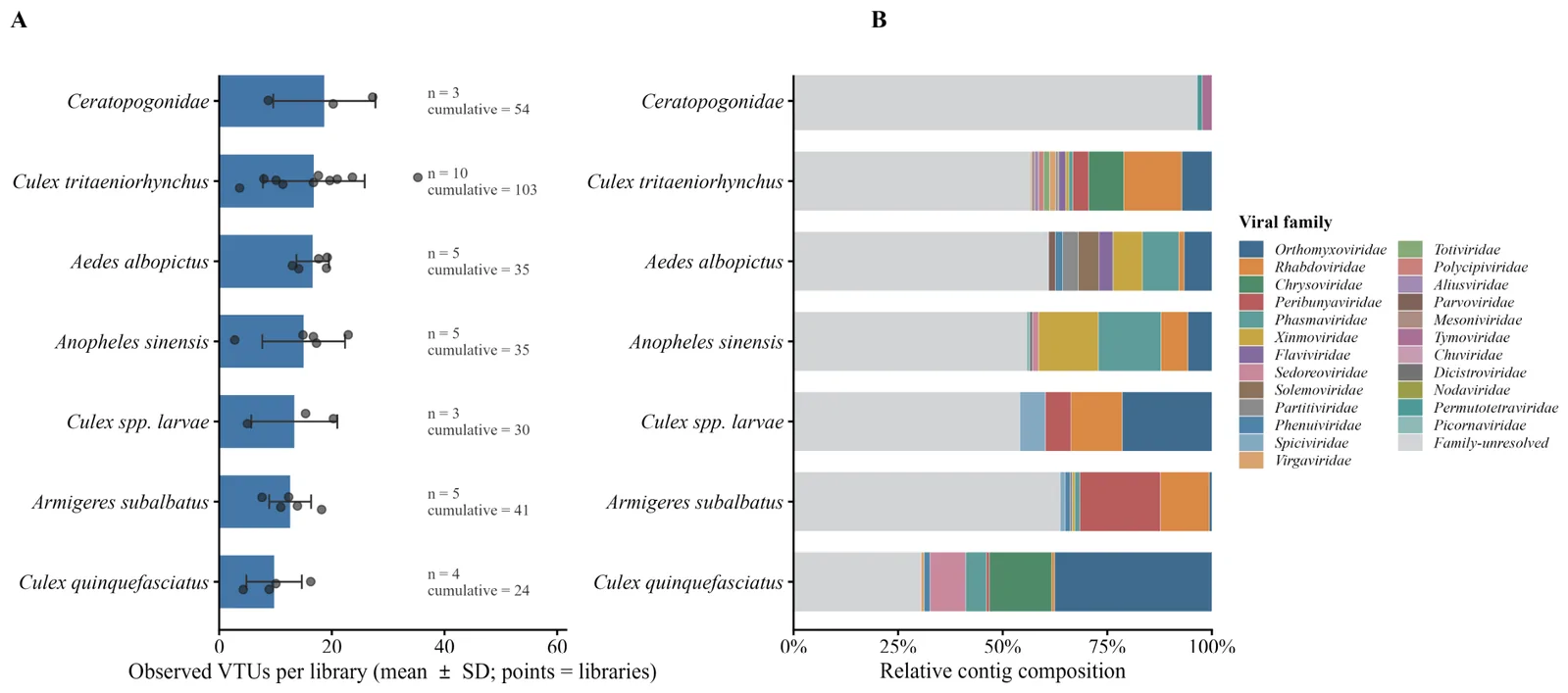
Host-group-associated viral richness and family composition. (**A**) Library-level observed VTU richness for each host group. Each point represents one library; bars show the mean, horizontal error bars show ± SD, n gives the number of libraries, and “cumulative” gives the number of distinct VTUs detected across all libraries in that host group. The plotted values are observed richness and were not standardized by rarefaction. (**B**) Relative composition of retained viral contigs by family within each host group. Each horizontal bar sums to 100% and includes all 24 assigned families plus the family-unresolved category. Richness comparisons are descriptive because the number of libraries and the sequencing depth differed between host groups. Means and SDs in panel A are descriptive, not inferential tests. Separate effort-standardized and depth-adjusted comparisons are reported in Figure S5 and Table S13.

*Cx. tritaeniorhynchus* had the highest observed cumulative richness, with 103 annotation-defined VTUs from 16 assigned families across 10 libraries (Figure 3A). The mean observed richness per library was highest in *Ceratopogonidae* (18.7 VTUs; three libraries), followed by *Cx. tritaeniorhynchus* (16.8; 10 libraries) and *Aedes albopictus* (16.6; five libraries). These rankings are descriptive because host-group replication was unequal and observed richness correlated positively with sequencing depth (Spearman ρ = 0.392, *p* = 0.020; Table S5). Rarefaction to 28,163,793 pre-depletion clean reads changed expected richness by <0.001 VTU per library, conditional on retained annotations. Equalizing effort to three libraries reduced the *Cx. tritaeniorhynchus* cumulative count to a median of 40.5 VTUs across 120 combinations (range, 17–71), versus 54 in the three *Ceratopogonidae* libraries. The depth-adjusted negative-binomial model did not detect an overall host-group richness difference (likelihood-ratio *p* = 0.412; Figure S5; Table S13). The family profiles also varied between host groups in the pooled summaries in Figure 3B and across individual host-group-by-location combinations (Figure S1).

### 3.3. Virome Discovery and Phylogenetic Placement

**Figure 4:**
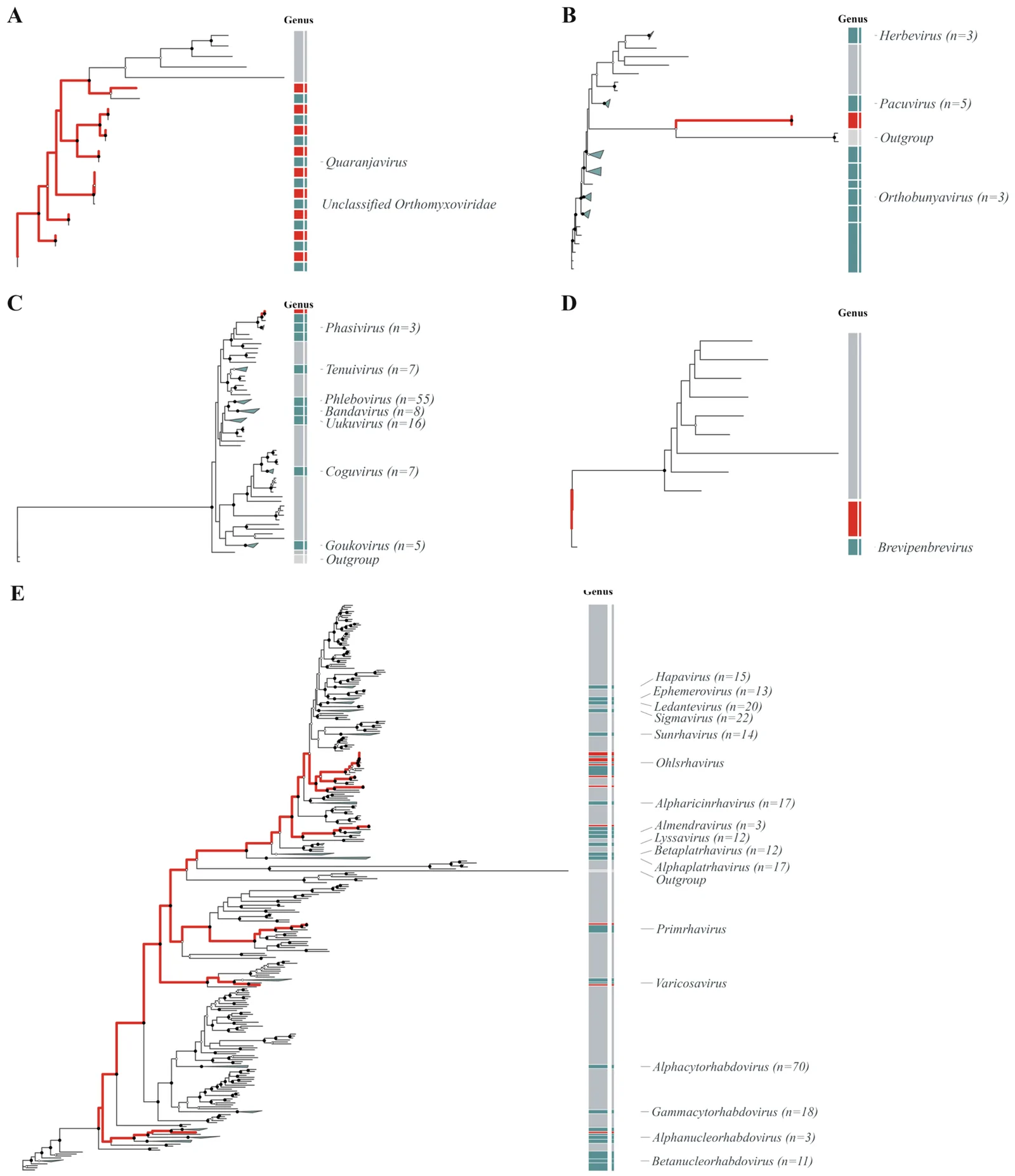
Maximum-likelihood phylogenies of five selected viral families: (**A**) *Orthomyxoviridae*, (**B**) *Peribunyaviridae*, (**C**) *Phenuiviridae*, (**D**) *Parvoviridae*, and (**E**) *Rhabdoviridae*. Red branches lead to sequences recovered from the Yunnan libraries; black branches represent references. Colored triangles denote collapsed pure-genus reference clades, and the aligned strips identify selected reference genera. Gray strips denote other, unassigned, or outgroup references. Terminal sequence names are omitted from the overview; sequence counts, alignment lengths, models, and SH-aLRT/UFBoot values are listed in Table S14. Complete Newick files, alignments, and tip-taxonomy metadata are provided in File S2. Filled and open node symbols indicate UFBoot support of 100% and 90–99%, respectively.

**Figure 5:**
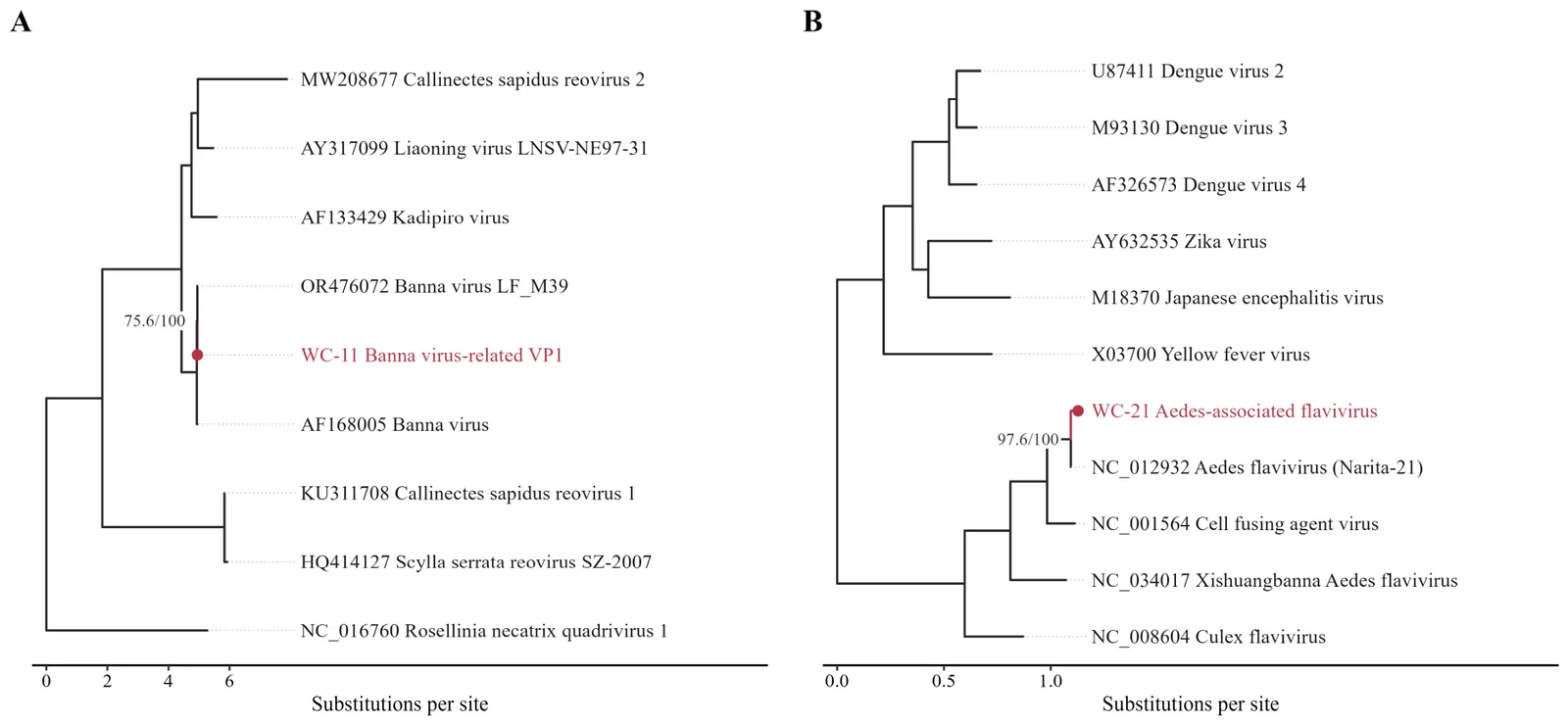
Targeted maximum-likelihood phylogenies of two representative detections. (**A**) Placement of the WC-11 Banna virus-related VP1 sequence after inclusion of LF_M39 VP1 (OR476072). (**B**) Placement of the WC-21 *Aedes*-associated flavivirus with representative insect-specific and vertebrate-infecting orthoflaviviruses. Red terminal branches and points mark sequences recovered in this study. Values beside the focal nodes are SH-aLRT/UFBoot percentages. Alignment lengths, substitution models, and patristic distances are provided in Table S15. The panels show selected-reference views of analyses containing 38 sequences in A and 107 in B; the complete trees are supplied in File S2. Reference selection was for display only.

**Figure 6:**
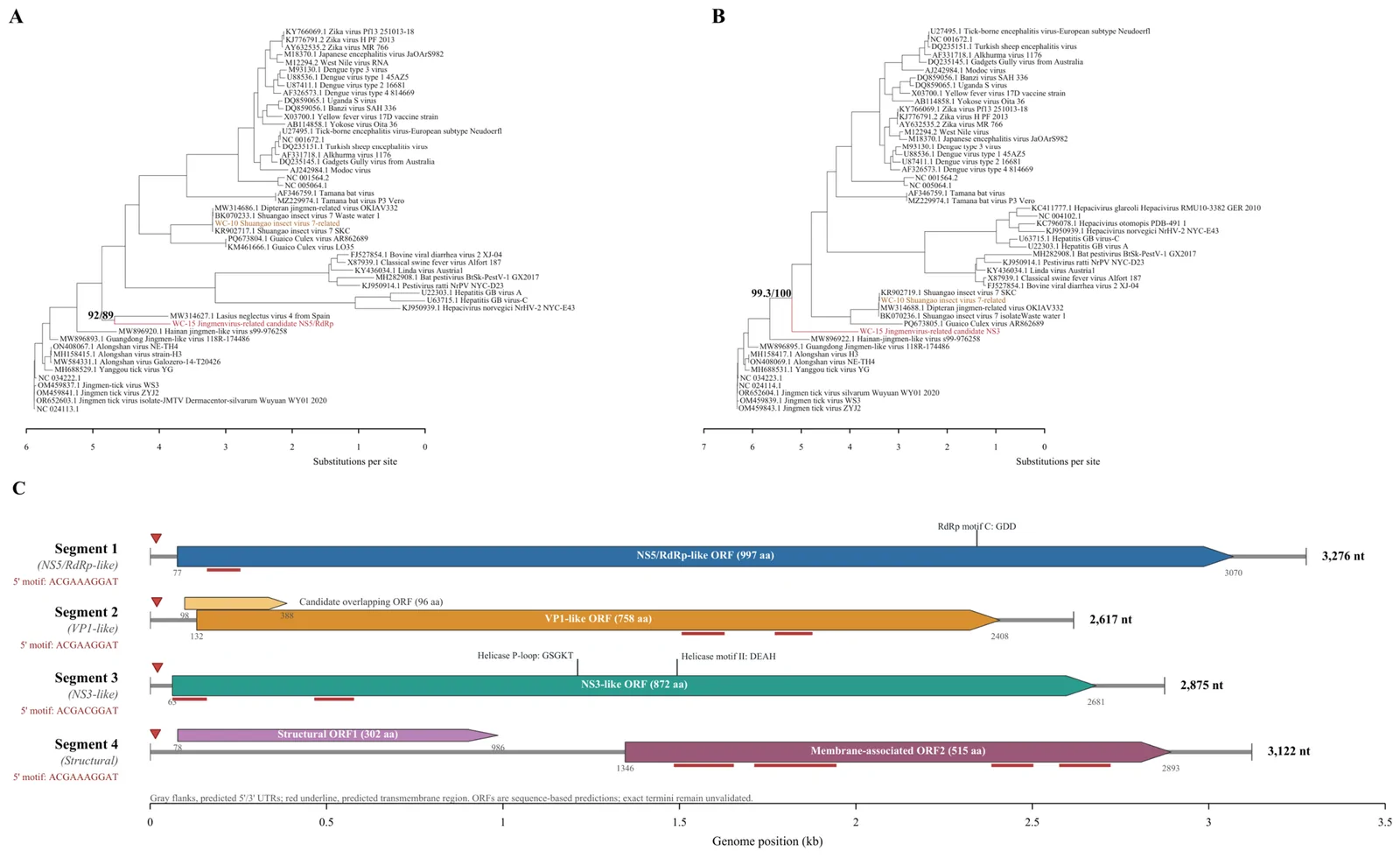
Phylogenetic placement and putative genome organization of WC-15 Jingmenvirus-related sequences. (**A**) Full NS5/RdRp and (**B**) full NS3 phylogenies, each containing 50 sequences. Values beside the WC-15 nodes denote SH-aLRT/UFBoot support (%); branch lengths are in substitutions per site. The WC-15 sequences are shown in red. (**C**) Provisional four-component organization based on sequence annotation. Arrows indicate predicted ORFs, gray flanks denote assembled terminal non-coding regions, red underlines indicate predicted transmembrane regions, and triangles mark related 5′ motifs. The principal ORFs, a candidate overlapping ORF, and selected RdRp and helicase motifs are shown. Component association and complete termini remain to be validated. Tree files are provided in File S2.

Five families were selected for the phylogenetic overview: *Orthomyxoviridae*, *Peribunyaviridae*, *Phenuiviridae*, *Parvoviridae*, and *Rhabdoviridae* (Figure 4). Selection was based on their representation among retained contigs, their relevance to arthropod-associated virus surveillance [36,37], and the availability of homologous polymerase or NS1 regions suitable for family-level placement. The five alignments contained 23, 122, 142, 12, and 539 sequences, including 9, 2, 1, 2, and 9 study-derived sequences from the 35 analyzed libraries, respectively. Selected models and individual study-sequence support values are reported in Table S14. These five families represent 5 of the 24 assigned families. The Banna VP1 tree is shown in Figure 5A, the *Aedes*-associated flavivirus tree in Figure 5B, and the WC-15 NS5/RdRp and NS3 trees in Figure 6A,B. Selected genus labels summarize reference clades and do not assign every recovered sequence to a genus.

#### 3.3.1. Overview of Representative Detections

The representative detections ranged from close relatives of known viruses to remote protein-level homologs (Table 1). Their sequence identities, evidence classes, and taxonomic interpretations are summarized below, and results are discussed in detail in subsequent sections.

#### 3.3.2. Banna Virus-Related Sequences

Twelve WC-11 contigs were assigned to Banna virus VP1–VP12, providing sequence signals for each of the 12 genome segments (Table S9). VP1 was represented by a relatively complete 3867 nt assembly and had by far the strongest read support (1,394,956 remapped reads); it was therefore selected for phylogenetic analysis. The other segment-associated contigs ranged from 860 to 3071 nt and were supported by 1611–25,211 remapped reads. All 12 assignments had nucleotide-level matches to Banna virus references, with 97.28–99.65% nucleotide identity. VP1 clustered within the established Banna virus lineage and grouped directly with strain LF_M39 (OR476072; SH-aLRT/UFBoot = 75.6/100; patristic distance, 0.00714 substitutions per site), isolated from *Anopheles sinensis* in Lufeng, Yunnan [38] (Figure 5A; Table S15). Its best nucleotide match to LF_M39 segment 1 had 99.2% identity.

#### 3.3.3. *Aedes*-Associated Flavivirus

The WC-21 sequence recovered from *Aedes albopictus* grouped directly with *Aedes* flavivirus strain Narita-21 (NC_012932; SH-aLRT/UFBoot = 97.6/100) in the focused tree (Figure 5B; Table S15). This clade also contained Cell fusing agent virus and other insect-specific viruses placed within the broader orthoflavivirus radiation [39]. Its phylogenetic placement and nucleotide and amino-acid identities support its assignment as a close relative of a known insect-specific flavivirus rather than as a deeply divergent lineage.

#### 3.3.4. Divergent Jingmenvirus-Related Sequences

The WC-15 *Ceratopogonidae* library from Lushui contained four Jingmenvirus-related coding sequences, corresponding to NS5/RdRp-like, VP1-like, NS3-like, and structural proteins. The NS5/RdRp and NS3 proteins shared 34.9% identity across 920 amino acids (92.3% of the predicted 997-aa ORF) and 29.4% identity across 688 amino acids (78.9% of the predicted 872-aa ORF), respectively, with their closest protein-level match, Lasius neglectus virus 4. They occupied long, distinct branches; their immediate placement nodes had SH-aLRT/UFBoot support of 92/89 and 99.3/100, respectively (Figure 6A,B; Table 1; Table S15). The four assemblies were 3276, 2617, 2875, and 3122 nt, totaling 11,890 nt. Their functional assignments match the four-component organization described for members and close relatives of the genus Jingmenvirus [39,40,41,42,43,44,45,46]: an NS5/RdRp component, an NS3 protease-helicase component, a VP1-like component, and a component carrying a structural ORF1 and a membrane-associated ORF2 (Figure 6C). The candidate grouping follows comparative criteria used in the original description of Jingmen tick virus, particularly complementary coding functions and related segment termini [40]. Segment-specific remapping in WC-15 yielded 34.1, 30.9, 36.6, and 80.9 RPM for Segments 1–4, respectively, approximating a 1:1:1:2 profile (Table S6). After normalization by assembled length, the relative profile was 0.89:1.01:1.09:2.22 (Table S6). Segment 4 remained relatively enriched. RPM and length-normalized values depend on mapping and assembly properties and are supporting context, not molar ratios. Similar enrichment of Segment 4 has been reported in independent Jingmenvirus-related libraries [47,48]. Complementary coding functions, related assembled 5’ motifs, and independent read remapping support treating the four RNAs as putative components of a candidate WC-15 Jingmenvirus-related genome (Tables S6, S7, and S10; Figure S2; File S3). The predicted organization included the principal ORFs, a short overlapping ORF in the VP1-like sequence, two structural ORFs, the RdRp GDD motif, the NS3 helicase P-loop and DEAH motifs, and several predicted transmembrane regions (Figure 6C). Physical linkage among the components has not been demonstrated.

### 3.4. Ecological Structure Across Host Groups, Life Stages, and Regions

#### 3.4.1. Host-Group and Site-Level Relationships

**Figure 7:**
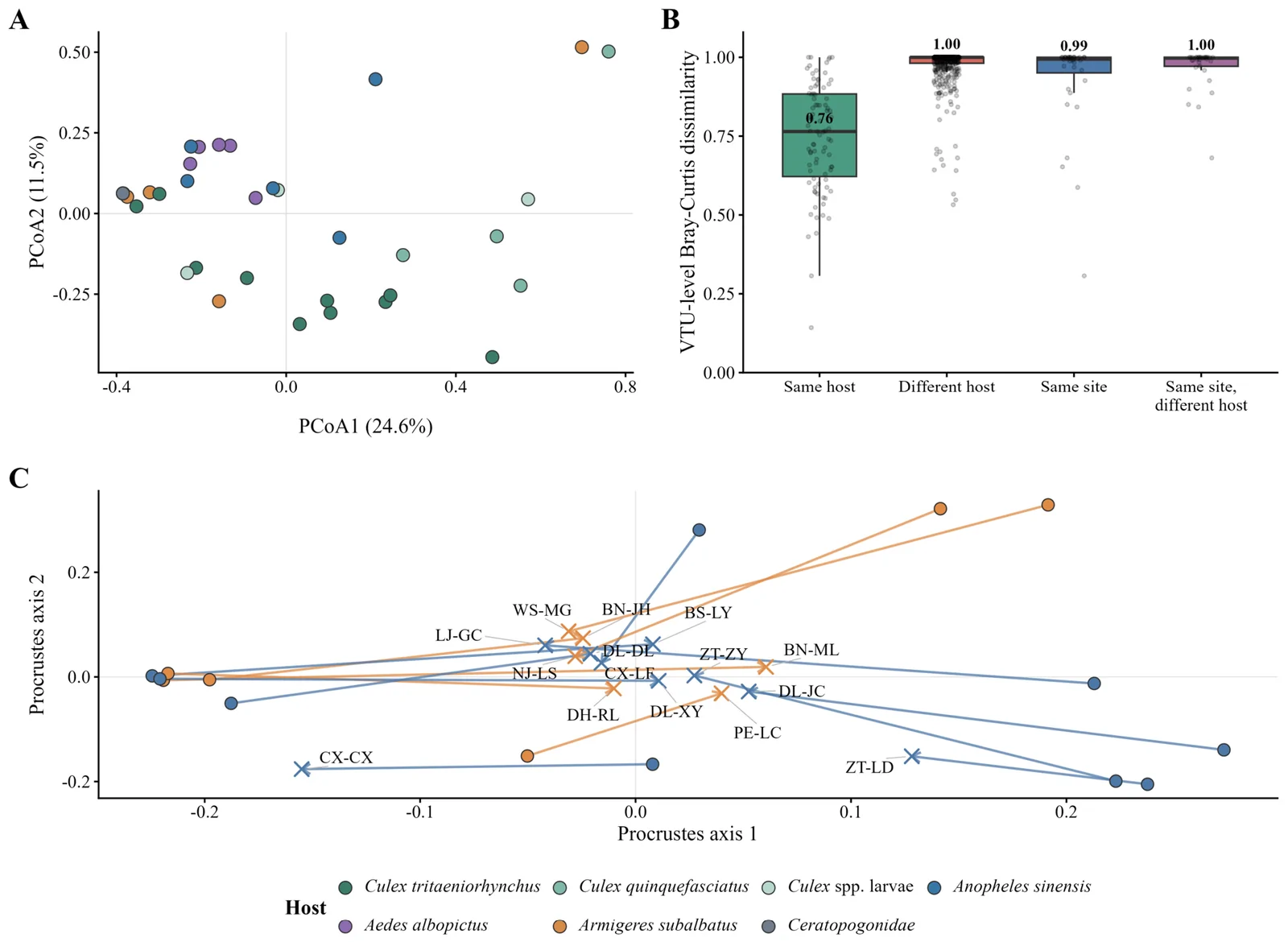
Community structure across sampling scales. (**A**) PCoA of family-level viromes in 35 pooled libraries. (**B**) VTU-level Bray-Curtis dissimilarity between library pairs; medians are displayed on the boxes. (**C**) Procrustes comparison of site-level host-group (circles) and virome (crosses) configurations at 15 matched locations; connecting segments show residual mismatch. Blue and orange denote the author-defined plateau and subtropical groups, respectively. Pairwise distributions are descriptive because libraries occur in multiple pairs.

The host-group term explained 36.7% of the Bray-Curtis variation between the 35 family-level profiles (PERMANOVA, *p* < 0.001; Figure 7A). Restricting permutations to within the sampling location produced the same effect-size estimate (*p* < 0.001). After adjustment for location and sequencing depth, the marginal host-group term explained 21.3% of the variation (*p* = 0.0052). Same-host-group library pairs were less dissimilar than different-host-group pairs (median Bray-Curtis dissimilarity, 0.765 versus 1.000), whereas different host groups sampled at the same site remained highly dissimilar (median, 0.997; Figure 7B).

The host-group association persisted across alternative VTU definitions, evidence filters, and exclusion of single-contig VTUs. In the seven location- and depth-adjusted sensitivity tests, the host-group term explained 26.3–29.0% of variation (all BH-adjusted q = 0.0001; Figure S3; Tables S3 and S17). The association also persisted in models with permutations restricted within location in all 35 libraries (R^2^ = 0.213, *p* = 0.0004), the 28 libraries from multi-host locations (R^2^ = 0.261, *p* = 0.0009), and the 32 averaged host-location profiles (R^2^ = 0.218, *p* = 0.0056; Table S4). PERMDISP indicated possible unequal within-group dispersion (F = 2.45, permutation *p* = 0.0489; Figure S4B). The *p*-value was 0.0850 after small-sample and Lingoes adjustments (Table S12). The host-associated separation may therefore reflect both compositional differences and differences in within-group heterogeneity.

At the 15 sequenced locations, host-group and virome configurations showed no significant Procrustes concordance (r = 0.369, *p* = 0.295; Figure 7C). Site-level host counts, virome profiles, and Procrustes results are provided in Table S19.

#### 3.4.2. Adult-Larval Comparison

**Figure 8:**
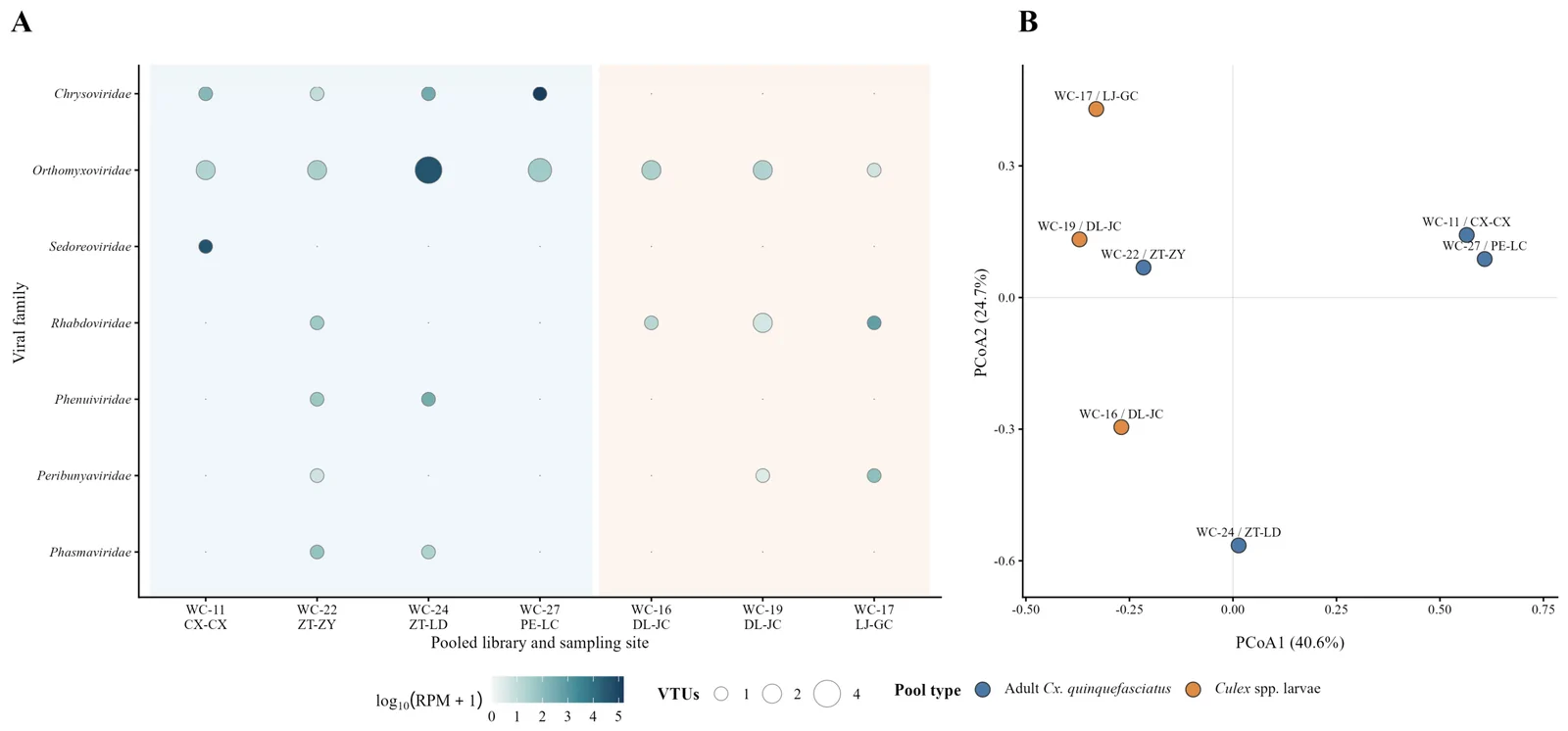
Viromes associated with adult and larval *Culex* pools. (**A**) Viral family profiles for four adult *Culex quinquefasciatus* libraries and three *Culex* spp. larval libraries; bubble area represents VTU count and color represents log_10_(RPM + 1). (**B**) PCoA of family-level composition, with library and site labels.

Four adult *Culex quinquefasciatus* libraries and three *Culex* spp. larval libraries contained 46 annotation-defined VTUs. Sixteen VTUs (34.8%) occurred only in adult pools, eight (17.4%) were shared, and twenty-two (47.8%) occurred only in larval pools. *Chrysoviridae* and *Orthomyxoviridae* were detected in all four adult libraries, and high *Sedoreoviridae* abundance was restricted to WC-11. *Orthomyxoviridae* and *Rhabdoviridae* occurred in all three larval libraries, whereas *Peribunyaviridae* occurred in a subset (Figure 8A). The family-level ordination provided no evidence of a stage-associated difference (PERMANOVA, R^2^ = 0.266, exact *p* = 0.143; Figure 8B), and PERMDISP yielded *p* = 0.057. Because developmental stage, taxonomic resolution, and location were confounded, this comparison describes differences between the sampled adult and larval pools rather than a within-species developmental effect.

#### 3.4.3. Spatial Turnover Within *Cx. tritaeniorhynchus*

**Figure 9:**
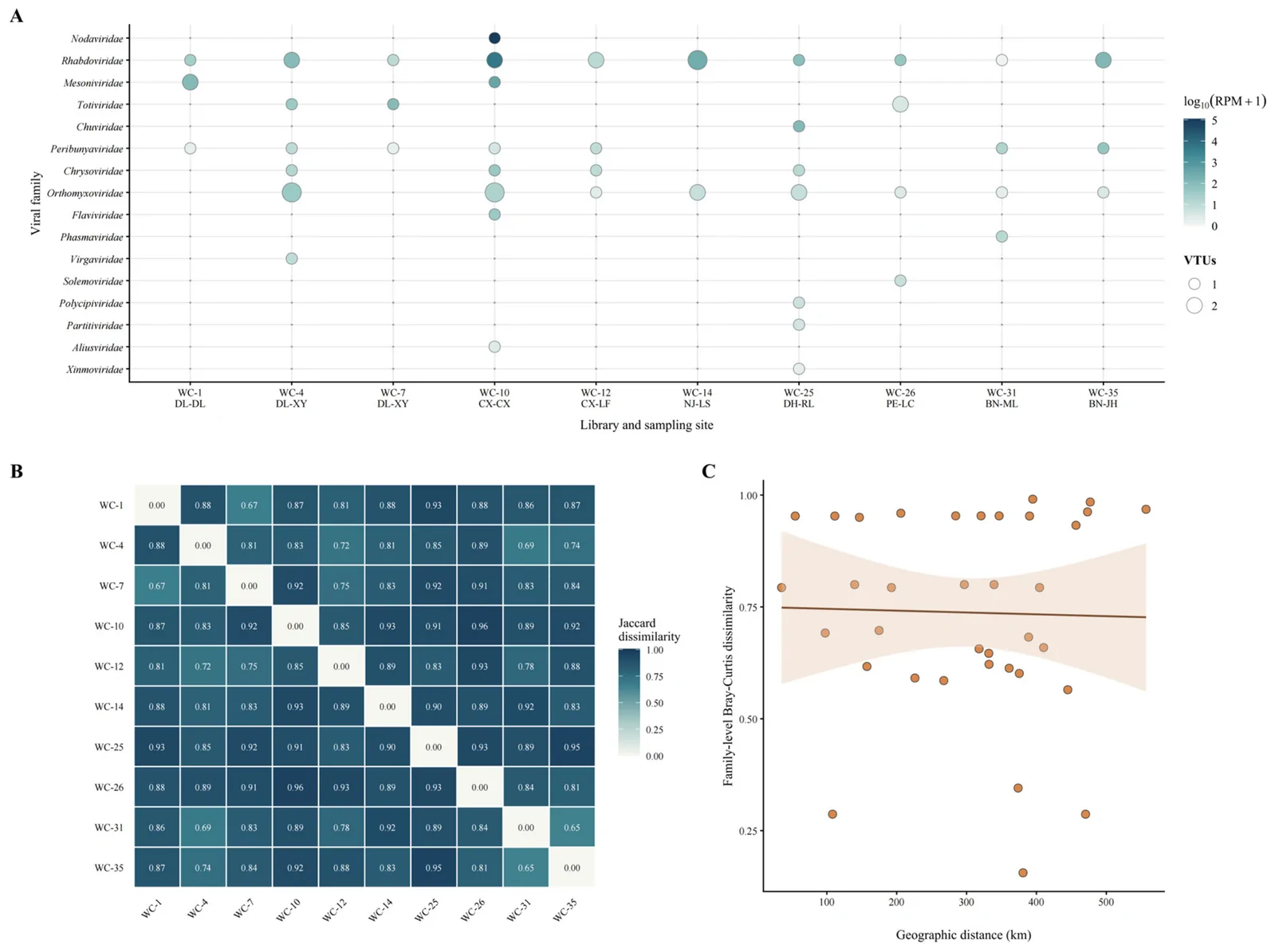
Spatial turnover within *Cx. tritaeniorhynchus*. (**A**) Viral family profiles for 10 libraries from nine locations; bubble area represents VTU count and color represents log_10_(RPM + 1). All assigned families detected in this host are shown. (**B**) Pairwise binary Jaccard dissimilarity between libraries. (C) Geographic distance and family-level Bray–Curtis dissimilarity between the nine sampled locations; the line and band show the linear fit and its 95% confidence interval, respectively. The fitted line is descriptive; the geographic association was tested by the Spearman Mantel test reported in the text.

*Cx. tritaeniorhynchus* allowed the largest within-host-group comparison, with 10 libraries from nine locations. VTU richness ranged from 8 in Dali to 35 in Chuxiong. Ruili, Xiangyun, and Lancang contained 24, 21, and 20 VTUs, respectively. Of the 103 VTUs detected in this host group, 77 (74.8%) were detected at one sampled site, 21 (20.4%) at two to four sites, and five (4.9%) at five or more sites. The median binary Jaccard dissimilarity between the libraries was 0.872 (Figure 9B). Single-site detections remained common after nucleotide-only filtering (42/54 VTUs, 77.8%) or a ≥20-read filter (75.0%). Retaining either one of the two DL-XY libraries yielded 74.8–78.8% single-site detections across nine libraries (Table S16). The family profiles also varied between locations (Figure 9A), but no significant monotonic association between geographic distance and family-level dissimilarity was detected (Spearman Mantel ρ = 0.089, *p* = 0.298; Figure 9C). These summaries describe observed detections, not true site occupancy or geographic restriction.

#### 3.4.4. Broad Climate-Zone Patterns

**Figure 10:**
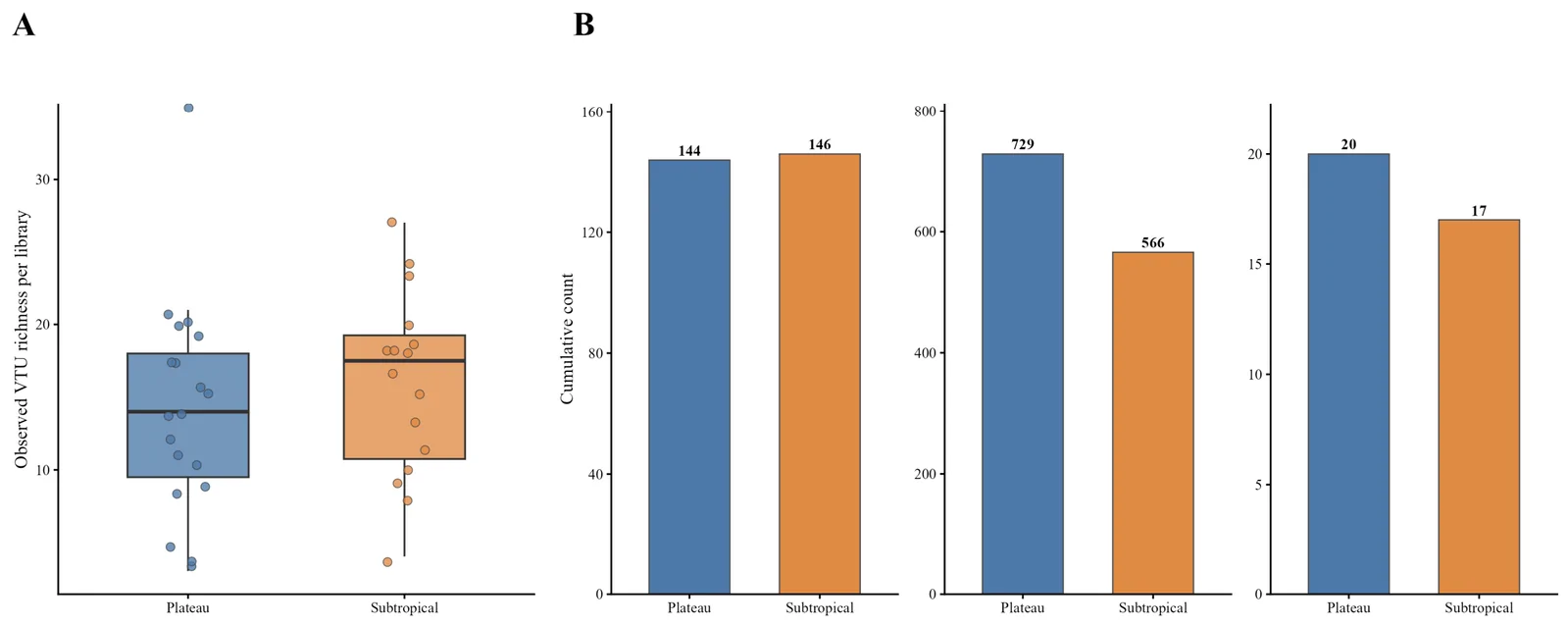
Virome diversity in two broad Yunnan climate-zone groups. (**A**) Observed VTU richness per library in the plateau cool/moderate and subtropical hot/wet groups. (**B**) Cumulative annotation-defined VTUs, viral contigs, and assigned families in each group; each facet has its own vertical scale. No statistically significant difference in library-level richness was detected (Wilcoxon *p* = 0.371).

The author-defined classification assigned the 15 sequenced locations to two broad Yunnan climate-zone groups: nine plateau cool/moderate sites (19 libraries) and six subtropical hot/wet sites (16 libraries). The median observed richness was 14 VTUs per library in the plateau group (IQR, 9.5–18.0) and 17.5 in the subtropical group (IQR, 10.8–19.2; Wilcoxon *p* = 0.371). The cumulative VTU richness was similar between groups (144 versus 146), whereas the plateau group contained more contigs (729 versus 566) and more assigned families (20 versus 17). No single climate-zone group ranked higher for every diversity measure (Figure 10).

## 4. Discussion

The pooled libraries broaden the viral sequence record for mosquitoes and biting midges in Yunnan. The predominance of insect-associated viruses, partial assemblies, and family-unresolved sequences resembles findings from large metatranscriptomic surveys of mosquitoes and other invertebrates [3,4,5,29,36,37]. Direct richness comparisons between studies are limited by differences in sampling and annotation. This recurring pattern reflects the evolutionary breadth of arthropod viruses and their sparse representation in reference databases. The 221 annotation-defined VTUs provide a reproducible summary of the diversity recovered from these pools while retaining links to the underlying contigs. The combination of recognized viral lineages and highly divergent sequences shows that the regional virome contains established surveillance targets as well as poorly characterized diversity.

Among the surveillance-relevant findings, the Banna virus sequences merit particular attention. WC-11 contained sequences corresponding to each of the 12 genome segments, and VP1 was comparatively complete and closely related to strain LF_M39 from *Anopheles sinensis* in Lufeng [38]. Banna virus has repeatedly been isolated or detected in mosquitoes, biting midges, and ticks in Yunnan and Southeast Asia [38,49,50]. Banna virus has also been detected in clinical material from febrile or encephalitic patients [49,50]. Its detection in a *Culex quinquefasciatus* pool extends this regional sequence record but does not establish a clinical association for the present finding (Table S18). The close VP1 relationship to a local strain is consistent with continued regional occurrence of Banna virus-related viruses and provides a defined target for confirmatory amplification and isolation.

WC-15 represents a different type of finding. Its four coding components were only distantly related to known Jingmenviruses, yet their gene content matched the characteristic nonstructural and structural complement described for this group [40]. The related terminal motifs and independent read remapping support their interpretation as putative components of a candidate genome, without establishing physical linkage. Abundance provides supplementary context, not independent evidence of genome membership. The relative enrichment of Segment 4 also resembles the unequal segment accumulation reported in other Jingmenvirus-related libraries [47,48]. This agreement is consistent with earlier observations but does not establish common genome membership. Human infection with Jingmen tick virus has been reported [51], as well as for Alongshan virus [52]. Those reports do not establish the biological properties of WC-15. Conserved motifs support functional annotation, not an inference of virulence (Table S18). For WC-15, the main contribution is its marked phylogenetic divergence and detection in a *Ceratopogonidae* pool. The lineage adds sequence information to a poorly sampled part of the Jingmenvirus-related phylogeny and broadens the arthropod context in which these viruses have been detected. Isolation and validation of complete segment termini are now needed to resolve its taxonomic position and biological properties.

The high cumulative richness in *Cx. tritaeniorhynchus* requires a different interpretation. This host group also had more libraries and broader geographic representation than the others. Its cumulative ranking changed when library number was equalized, and many of its detected VTUs were insect-associated or unresolved. The species remains epidemiologically important for a different reason: it is a principal vector of Japanese encephalitis virus in Asia and participates in transmission cycles involving pigs and ardeid birds [18,53]. Surveillance in *Cx. tritaeniorhynchus* is therefore most informative when individual viral lineages are evaluated in relation to known vertebrate-host and vector associations, rather than when total richness is used as a risk measure.

Community composition provides a broader ecological context for these virus-level findings. The libraries from the same host group were more similar than libraries from different host groups, and this pattern remained after accounting for location, sequencing depth, and alternative VTU definitions. Comparable host-associated structure has been reported in sympatric, longitudinal, and geographically broad mosquito surveys [6,7,8,9,10,22,23]. Host taxonomy can integrate several processes that were not measured directly, including physiology, feeding behavior, habitat use, and the persistence of insect-associated viruses. Unequal within-group dispersion may contribute to this separation. Host group combines several unmeasured traits and should not be interpreted as a causal factor. The observed association should not be reduced to any single mechanism, but it shows that host composition must be considered when regional viromes are compared.

Local variation was pronounced within *Cx. tritaeniorhynchus*, but single-site detections can reflect incomplete detection, low abundance, and uneven sequencing depth. The nine-site Mantel test did not detect a monotonic distance-decay pattern; this does not exclude spatial structure. Temperature and precipitation associations remain unresolved because the application-display records lacked verified temporal and metric definitions. Larval habitat, hydrology, land use, and vegetation vary within sites [22,23,54]. One testable hypothesis is that viromes within a host group are more similar between sites with comparable larval habitats than between sites sharing only a broad climate-zone label. Land use and vertebrate-host availability are additional, unmeasured explanations. The limited overlap between adult *Culex quinquefasciatus* and *Culex* spp. larval pools describes the sampled pools because stage, taxonomic resolution, and location were confounded. Matched sampling across seasons and microhabitats will be needed to determine which host-associated patterns persist over time.

In addition, this study was limited by pooled sampling, uneven representation of host groups, incomplete genome recovery, and a cross-sectional collection in June 2025, which cannot resolve seasonal dynamics. Morphological identification may not distinguish cryptic taxa. Pooling can dilute low-abundance RNA and prevents attribution to individual insects. Reference-database gaps limit classification. No extraction or library-process negative controls were available; community-level sensitivity checks do not validate individual detections. Detection in pooled libraries does not establish infection, replication, or vector competence in the sampled arthropods. To address these limitations, balanced longitudinal sampling, improved host identification, targeted sequencing, and virus isolation should be performed in the future.

## 5. Conclusions

The 35 pooled libraries reveal a diverse and locally heterogeneous virome among Yunnan mosquitoes and biting midges. Sequences corresponding to Banna virus provide a defined target for regional surveillance, while WC-15 adds a divergent Jingmenvirus-related candidate represented by four putative components. Virome composition differed between host groups after sampling-design checks, and most VTUs in the best-sampled host were detected at one sampled site. These are community patterns in pooled libraries, not evidence of infection or transmission. Host-balanced longitudinal sampling and targeted isolation of the Banna virus and WC-15 candidates should be prioritized in future studies.

## Figures and Tables

**Table 1:** Sequence similarity and taxonomic interpretation of representative viruses. aa, amino acid; nt, nucleotide. The Banna virus identity refers to the VP1 nucleotide match.

Representative Virus	Host Pool/Location	Component	Closest Reference	Similarity	Evidence	Interpretation
Banna virus-related	*Culex quinquefasciatus*; WC-11; Chuxiong	VP1; 12 segment-like sequences	Banna virus	99.2% nt	Nucleotide-supported	Banna virus-related material; 12 segment-like sequences co-detected
*Aedes*-associated flavivirus	*Aedes albopictus*; WC-21; Dali	Polyprotein regions	*Aedes* flavivirus	96.0–99.7% aa; ~99.9% nt	Nucleotide-supported	Close relative of a known insect-associated flavivirus
WC-15 Jingmenvirus-related candidate	*Ceratopogonidae*; WC-15; Lushui	NS5/RdRp	*Lasius neglectus* virus 4	34.9% aa over 920 aa; 92.3% query coverage	Protein-supported	Divergent candidate lineage
WC-15 Jingmenvirus-related candidate	*Ceratopogonidae*; WC-15; Lushui	NS3	*Lasius neglectus* virus 4	29.4% aa over 688 aa; 78.9% query coverage	Protein-supported	Divergent candidate lineage

## Data Availability

The raw sequencing data generated in this study are publicly available in the Genome Sequence Archive (GSA) under accession CRA048980 (BioProject PRJCA072712). Viral nucleotide sequences reported in this study have been deposited in GenBank under accession numbers PZ933790–PZ933827.

## Supplementary Materials

The following supporting information can be downloaded at: https://www.mdpi.com/article/10.3390/v18101109/s1, Supplementary Methods S1. Ecological, statistical, read-rarefaction, and terminal-motif procedures, including weather-record and climate-zone definitions; Table S1. Library and location metadata; Table S2. The 221 annotation-defined VTUs and their operational definition; Tables S3 and S4. Host-group and design sensitivity analyses, including BH-adjusted *p*-values; Table S5. Sequencing-depth diagnostics; Tables S6, S7, S9 and S10. WC-15 and Banna virus sequence support; Table S8. Field specimen counts and sampling-site context; Table S11. Site-by-host sampling overlap; Table S12. PERMDISP distances and sensitivity checks; Table S13. Equal-library, depth-adjusted, and read-rarefied richness; Table S14. Family-level tree parameters and study-sequence support; Table S15. Focused phylogeny results; Table S16. Spatial detection and occupancy summaries under evidence and sampling filters; Table S17. Single-contig VTU summaries, exclusion sensitivity, and seven-test BH correction; Table S18. Evidence-based interpretation of representative detections; Table S19. Application-display record and climate-zone definitions, site-level inputs, and Procrustes results; Figures S1–S6. Library profiles, WC-15 terminal motifs, host-group robustness, sampling overlap, multivariate dispersion, richness sensitivity, and full WC-15 trees; File S1. Retained viral contig sequences; File S2. Alignments, phylogenies, model reports, and tip metadata; File S3. WC-15 sequence and read-support files.

## Abbreviations

The following abbreviations are used in this manuscript:

**Abbreviation**	**Definition**
aa	amino acid
VTU	viral taxonomic unit
BN-JH	Jinghong City, Xishuangbanna Dai Autonomous Prefecture
BN-ML	Mengla County, Xishuangbanna Dai Autonomous Prefecture
bp	base pair
BS-LY	Longyang District, Baoshan City
CX-CX	Chuxiong City, Chuxiong Yi Autonomous Prefecture
CX-LF	Lufeng City, Chuxiong Yi Autonomous Prefecture
DH-RL	Ruili City, Dehong Dai and Jingpo Autonomous Prefecture
DL-DL	Dali City, Dali Bai Autonomous Prefecture
DL-JC	Jianchuan County, Dali Bai Autonomous Prefecture
DL-XY	Xiangyun County, Dali Bai Autonomous Prefecture
IQR	interquartile range
LJ-GC	Gucheng District, Lijiang City
NCBI	National Center for Biotechnology Information
NJ-LS	Lushui City, Nujiang Lisu Autonomous Prefecture
nt	nucleotide
ORF	open reading frame
PCoA	principal coordinates analysis
PE-LC	Lancang Lahu Autonomous County, Pu’er City
PERMANOVA	permutational multivariate analysis of variance
PERMDISP	permutational analysis of multivariate dispersion
RdRp	RNA-dependent RNA polymerase
RPM	reads per million
rRNA	ribosomal RNA
SD	standard deviation
SH-aLRT	Shimodaira–Hasegawa approximate likelihood-ratio test
WS-MG	Maguan County, Wenshan Zhuang and Miao Autonomous Prefecture
ZT-LD	Ludian County, Zhaotong City
ZT-ZY	Zhaoyang District, Zhaotong City
