# Supplementary material for: Virome Diversity and Host-Associated Heterogeneity in Mosquitoes and Biting Midges Across Yunnan, China
Source: Viruses. 2026 Oct 8;18(10):1109. doi: 10.3390/v18101109 (PMC13709827; doi:10.3390/v18101109)

Viral-family detection and abundance across sequenced host-location cells

Each column represents one sequenced host-group-by-location cell; duplicate libraries are aggregated.

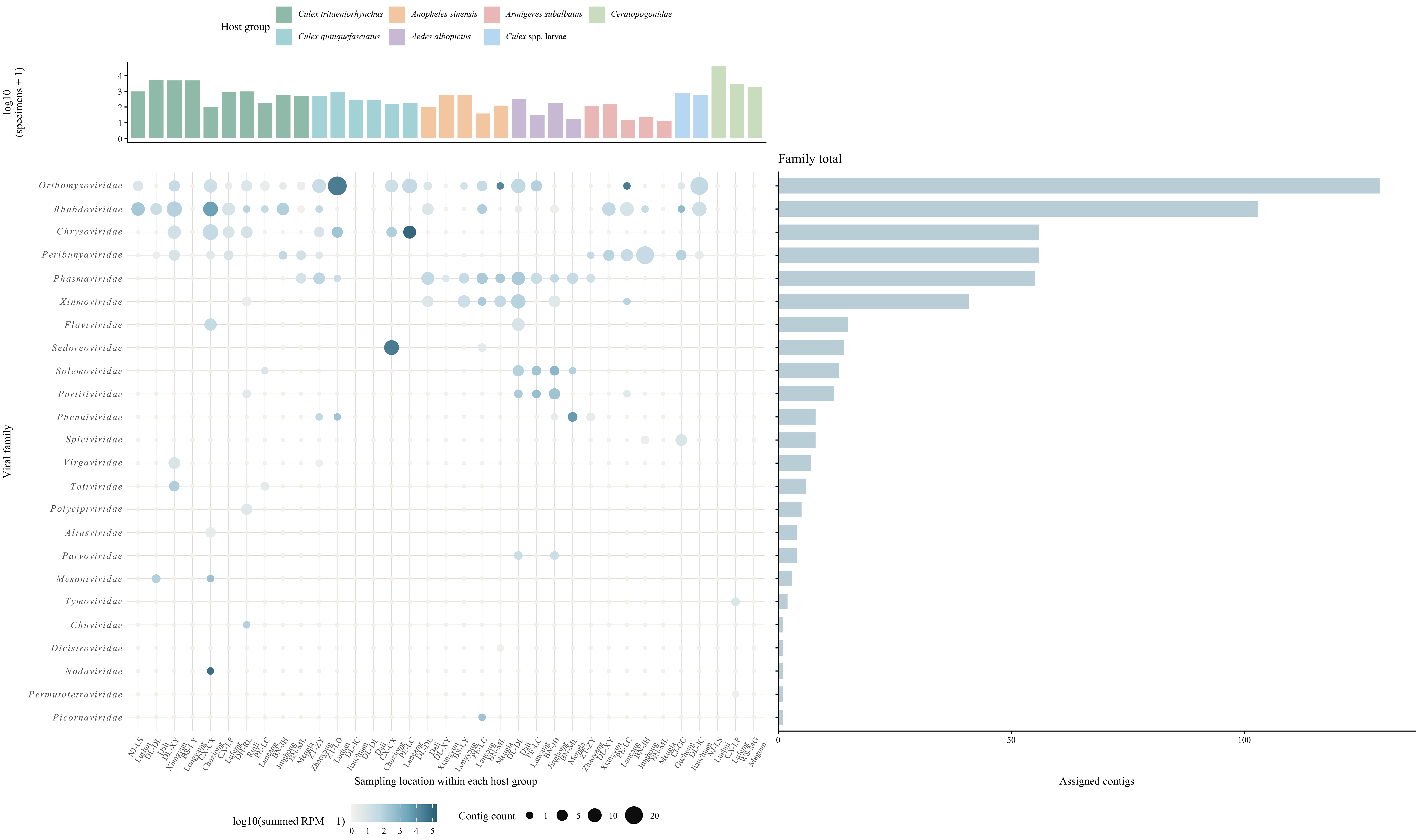

Supplement: Supplementary file 1 [file viruses-18-01109-s001.zip › FigureS1.pdf]
