# Supplementary material for: Virome Diversity and Host-Associated Heterogeneity in Mosquitoes and Biting Midges Across Yunnan, China
Source: Viruses. 2026 Oct 8;18(10):1109. doi: 10.3390/v18101109 (PMC13709827; doi:10.3390/v18101109)
Supplement: Supplementary file 1 [file viruses-18-01109-s001.zip › FigureS2.pdf]

Related 5' terminal motifs in the four WC-15 candidate segments

Segment	First 35 nt of the assembled sequence	Position
NS5	G G A C T T T A A G C C A A T **A C G A A A G G A T** T G A T G G A A T A	16-25
NS3	G T T T T T T T T A A T C A A C C A T **A C G A C G G A T** T T A G G T T	20-28
VP1-like	G T T T A A T C A T C T T A T A T **A C G A A G G A T** A G A A T T T G A	18-26
Structural	G T T T T A T A G C A T T **A C G A A A G G A T** A A A C C A C T A T A A	14-23

Highlighted bases match ACGA[AC]{1,2}GGAT within the first 40 nt. Positions are 1-based.

The comparison describes assembled 5' ends; exact genomic termini have not been experimentally verified.
