# Supplementary material for: Virome Diversity and Host-Associated Heterogeneity in Mosquitoes and Biting Midges Across Yunnan, China
Source: Viruses. 2026 Oct 8;18(10):1109. doi: 10.3390/v18101109 (PMC13709827; doi:10.3390/v18101109)
Supplement: Supplementary file 1 [file viruses-18-01109-s001.zip › FigureS3.pdf]

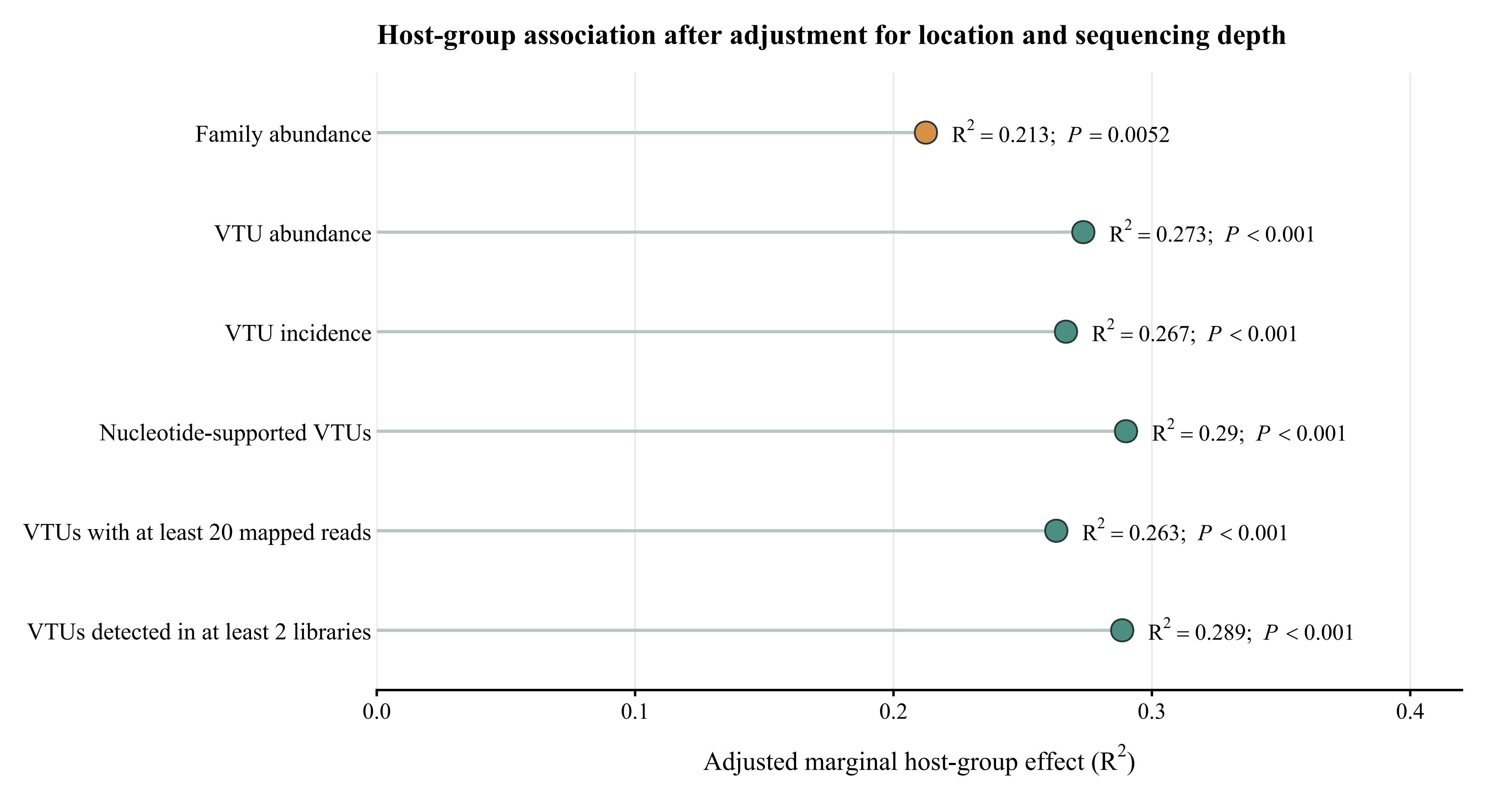

Host-group association after adjustment for location and sequencing depth
Family abundance
R2 = 0.213; P = 0.0052
VTU abundance
R2 = 0.273; P < 0.001
VTU incidence
R2 = 0.267; P < 0.001
Nucleotide-supported VTUs
R2 = 0.29; P < 0.001
VTUs with at least 20 mapped reads
R2 = 0.263; P < 0.001
VTUs detected in at least 2 libraries
R2 = 0.289; P < 0.001
0.0
0.1
0.2
0.3
0.4
Adjusted marginal host-group effect (R2)
