# Supplementary material for: Virome Diversity and Host-Associated Heterogeneity in Mosquitoes and Biting Midges Across Yunnan, China
Source: Viruses. 2026 Oct 8;18(10):1109. doi: 10.3390/v18101109 (PMC13709827; doi:10.3390/v18101109)
Supplement: Supplementary file 1 [file viruses-18-01109-s001.zip › FigureS4.pdf]

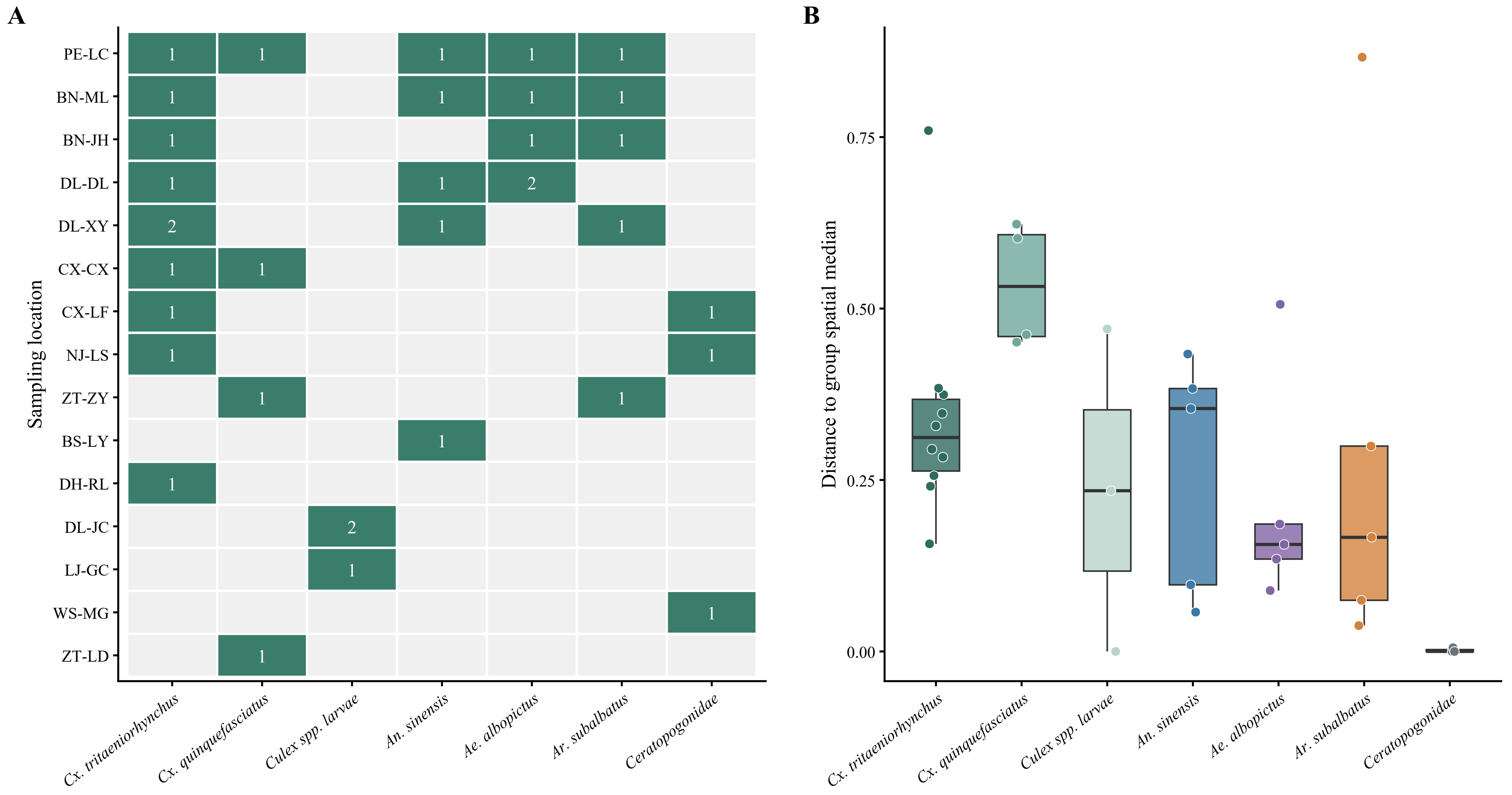
A
B
Sampling location
PE-LC
BN-ML
BN-JH
DL-DL
DL-XY
CX-CX
CX-LF
NJ-LS
ZT-ZY
BS-LY
DH-RL
DL-JC
LJ-GC
WS-MG
ZT-LD
Cx. tritaeniorhynchus
Cx. quinquefasciatus
Culex spp. larvae
An. sinensis
Ae. albopictus
Ar. subalbatus
Ceratopogonidae
Distance to group spatial median
0.00
0.25
0.50
0.75
