# Supplementary material for: Virome Diversity and Host-Associated Heterogeneity in Mosquitoes and Biting Midges Across Yunnan, China
Source: Viruses. 2026 Oct 8;18(10):1109. doi: 10.3390/v18101109 (PMC13709827; doi:10.3390/v18101109)
Supplement: Supplementary file 1 [file viruses-18-01109-s001.zip › FigureS5.pdf]

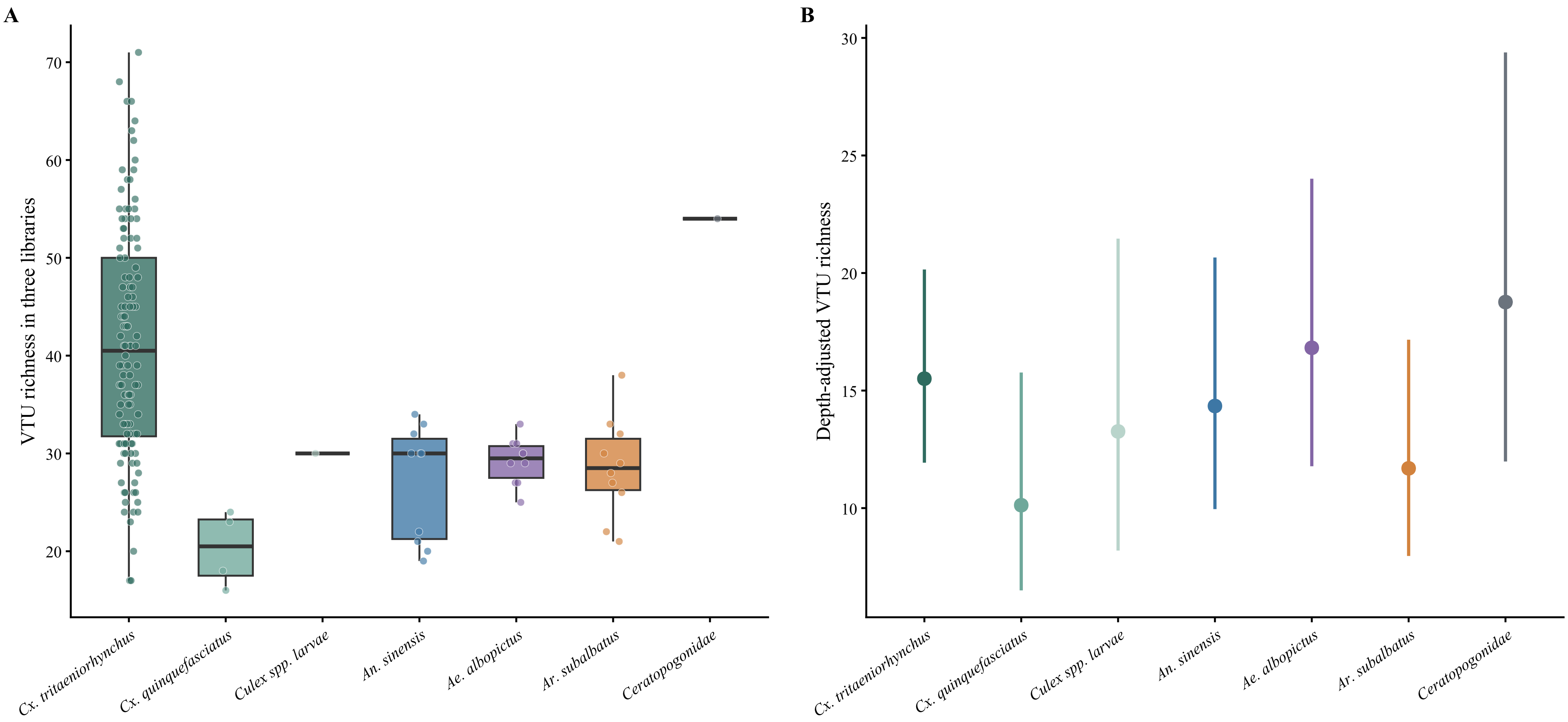
A
B
VTU richness in three libraries
Depth-adjusted VTU richness
70
60
50
40
30
20
30
25
20
15
10
Cx. tritaeniorhynchus
Cx. quinquefasciatus
Culex spp. larvae
An. sinensis
Ae. albopictus
Ar. subalbatus
Ceratopogonidae
