# Supplementary material for: Virome Diversity and Host-Associated Heterogeneity in Mosquitoes and Biting Midges Across Yunnan, China
Source: Viruses. 2026 Oct 8;18(10):1109. doi: 10.3390/v18101109 (PMC13709827; doi:10.3390/v18101109)
Supplement: Supplementary file 1 [file viruses-18-01109-s001.zip › FigureS6.pdf]

A

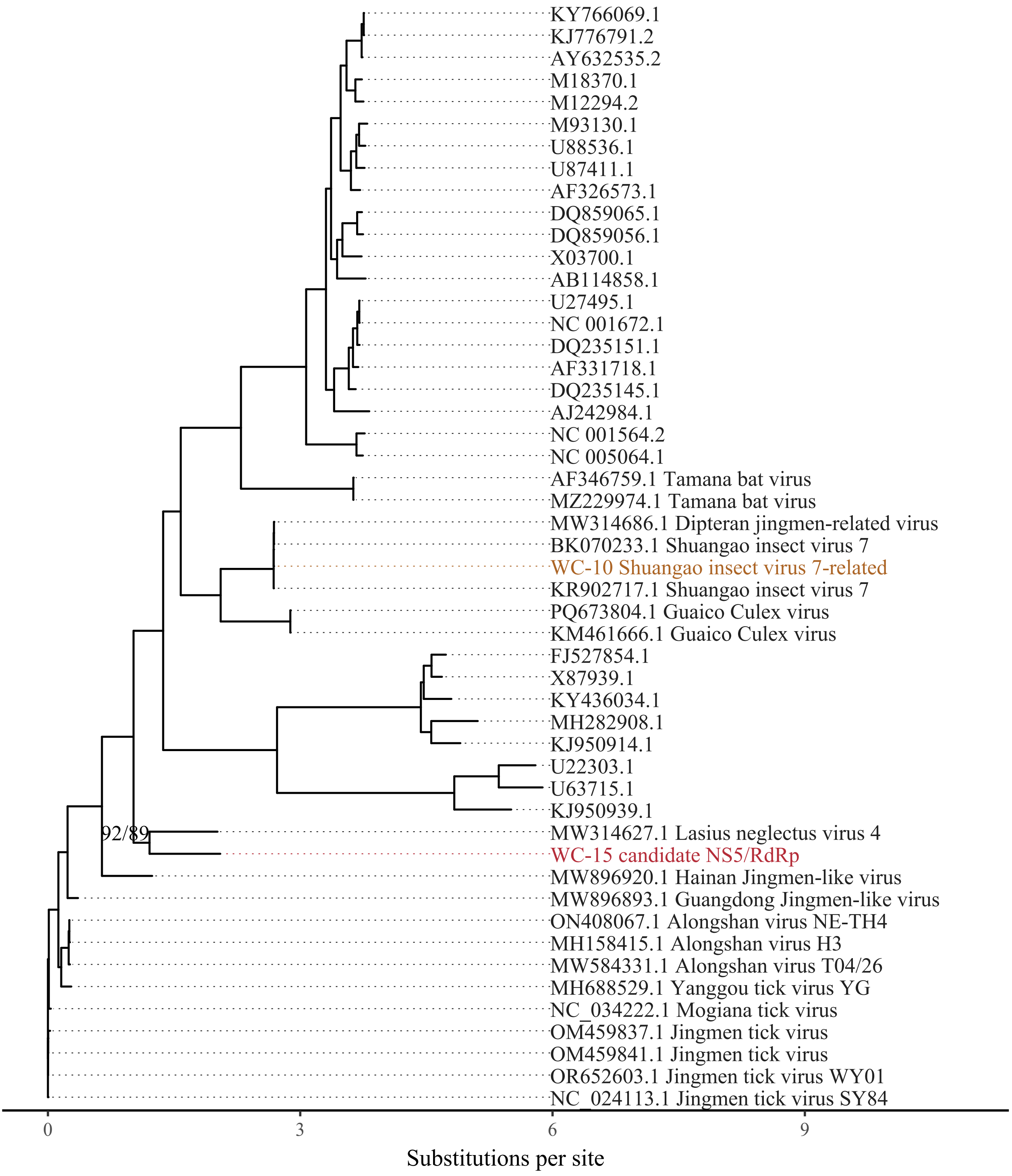

KY766069.1
KJ776791.2
AY632535.2
M18370.1
M12294.2
M93130.1
U88536.1
U87411.1
AF326573.1
DQ859065.1
DQ859056.1
X03700.1
AB114858.1
U27495.1
NC 001672.1
DQ235151.1
AF331718.1
DQ235145.1
AJ242984.1
NC 001564.2
NC 005064.1
AF346759.1 Tamana bat virus
MZ229974.1 Tamana bat virus
MW314686.1 Dipteran jingmen-related virus
BK070233.1 Shuangao insect virus 7
WC-10 Shuangao insect virus 7-related
KR902717.1 Shuangao insect virus 7
PQ673804.1 Guaico Culex virus
KM461666.1 Guaico Culex virus
FJ527854.1
X87939.1
KY436034.1
MH282908.1
KJ950914.1
U22303.1
U63715.1
KJ950939.1
92/89
MW314627.1 Lasius neglectus virus 4
WC-15 candidate NS5/RdRp
MW896920.1 Hainan Jingmen-like virus
MW896893.1 Guangdong Jingmen-like virus
ON408067.1 Alongshan virus NE-TH4
MH158415.1 Alongshan virus H3
MW584331.1 Alongshan virus T04/26
MH688529.1 Yanggou tick virus YG
NC_034222.1 Mogiana tick virus
OM459837.1 Jingmen tick virus
OM459841.1 Jingmen tick virus
OR652603.1 Jingmen tick virus WY01
NC_024113.1 Jingmen tick virus SY84
0
3
6
9
Substitutions per site

B

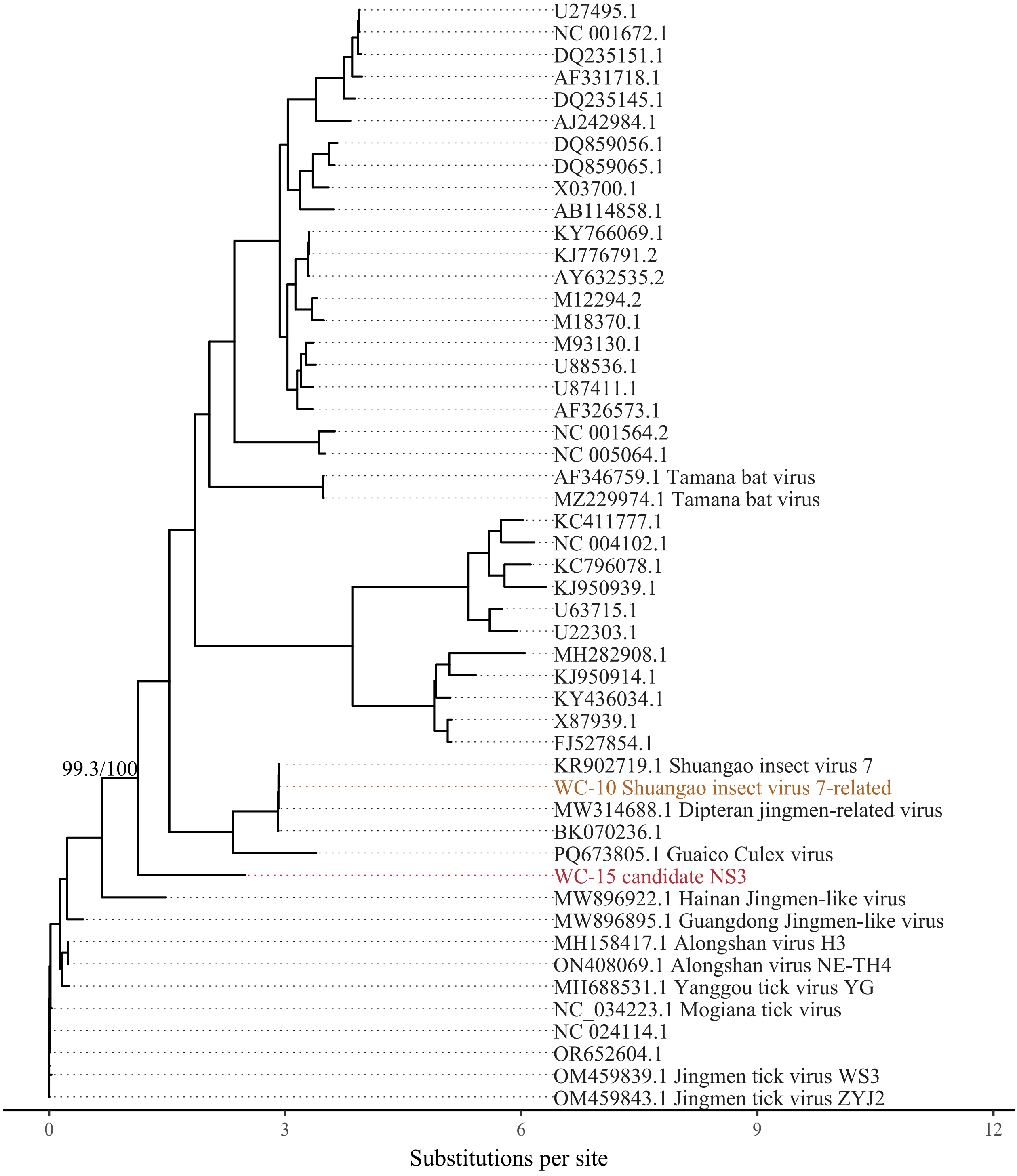

U27495.1
NC 001672.1
DQ235151.1
AF331718.1
DQ235145.1
AJ242984.1
DQ859056.1
DQ859065.1
X03700.1
AB114858.1
KY766069.1
KJ776791.2
AY632535.2
M12294.2
M18370.1
M93130.1
U88536.1
U87411.1
AF326573.1
NC 001564.2
NC 005064.1
AF346759.1 Tamana bat virus
MZ229974.1 Tamana bat virus
KC411777.1
NC 004102.1
KC796078.1
KJ950939.1
U63715.1
U22303.1
MH282908.1
KJ950914.1
KY436034.1
X87939.1
FJ527854.1
99.3/100
KR902719.1 Shuangao insect virus 7
WC-10 Shuangao insect virus 7-related
MW314688.1 Dipteran jingmen-related virus
BK070236.1
PQ673805.1 Guaico Culex virus
WC-15 candidate NS3
MW896922.1 Hainan Jingmen-like virus
MW896895.1 Guangdong Jingmen-like virus
MH158417.1 Alongshan virus H3
ON408069.1 Alongshan virus NE-TH4
MH688531.1 Yanggou tick virus YG
NC_034223.1 Mogiana tick virus
NC 024114.1
OR652604.1
OM459839.1 Jingmen tick virus WS3
OM459843.1 Jingmen tick virus ZYJ2
0
3
6
9
12
Substitutions per site
