# Supplementary material for: Virome Diversity and Host-Associated Heterogeneity in Mosquitoes and Biting Midges Across Yunnan, China
Source: Viruses. 2026 Oct 8;18(10):1109. doi: 10.3390/v18101109 (PMC13709827; doi:10.3390/v18101109)
Supplement: Supplementary file 1 [file viruses-18-01109-s001.zip › Supplementary_Methods_and_Figures.pdf]

viruses

MDPI

Supplementary Materials

Virome Diversity and Host-Associated Heterogeneity in Mosquitoes and Biting Midges across Yunnan, China

Honglin Su, Kai Zhao, Yuqiong Li, Zongti Shao, Xinglou Yang, Jian Wang, and Shengyao Chen

Supplementary Methods S1. Detailed ecological, statistical, and sequence analyses

S1.1. Sampling units, host groups, and duplicate cells

Host groups were defined at the finest level consistently available in the metadata: five adult *Culicidae* species, *Ceratopogonidae*, and *Culex spp.* larvae identified only to genus and developmental stage. Field records from the 15 locations contained 70,776 taxonomically resolved specimens in 35 host-group-by-location cells. Thirty-two cells contributed to sequencing. Three additional cells, *Culex quinquefasciatus* at DL-DL (300 specimens), *Culex quinquefasciatus* at DL-JC (280 specimens), and *Culex tritaeniorhynchus* at BS-LY (5,000 specimens), contributed to field host-composition summaries and site-level host analyses (Figure 1; Tables S8 and S19), but not to virome libraries.

The 35 libraries represented the 32 sequenced host-group-by-location cells. Three cells contained two libraries: *Aedes albopictus* at DL-DL (WC-3 and WC-21), *Culex tritaeniorhynchus* at DL-XY (WC-4 and WC-7), and *Culex spp.* larvae at DL-JC (WC-16 and WC-19). Primary library-level analyses retained these pools separately. A design-sensitivity analysis averaged family-level relative-abundance profiles within each duplicate cell before recalculating dissimilarities. The paired libraries arose within the same site, host group, and collection batch. The retained records do not establish whether the specimens in each pair were non-overlapping; the duplicate-cell sensitivity analysis therefore avoids treating those cells as independent site replicates.

Within each site, morphological host group, and collection batch, up to 50 specimens were randomly selected for each library; when fewer than 50 specimens were available, all were used. Pools were homogenized under cold conditions, and total RNA was extracted using the Virus DNA/RNA Extraction Kit 2.0 (RM01-04, Vazyme, Nanjing, China) according to the manufacturer's instructions. Libraries were prepared without rRNA depletion or poly(A) enrichment, were not strand-specific, and were sequenced as 150-bp paired-end reads on an Illumina NovaSeq 6000 platform.

S1.2. Community matrices and dissimilarities

RPM was calculated for each contig from reads mapped to the viral-contig reference divided by all quality-filtered reads in the same library before host and rRNA depletion, multiplied by one million. Family-level RPM values were summed across all 24 assigned viral families and the family-unresolved category, converted to relative abundance, and Hellinger-transformed. Bray-Curtis dissimilarity was calculated from the transformed matrix. VTUs followed the annotation-based definition in Methods 2.5: contigs with an identical curated BN_Species label were grouped across libraries, and their RPM values were added within a library. This was annotation-level consolidation, without a pairwise contig-identity threshold. A unit represented a reference-associated viral group and could include partial genes or genome segments. The annotation label was not itself evidence of strain identity or physical genome linkage. Table S2 records the definition and labels, and

Contig_to_VTU retains every input contig and its evidence class. Weighted VTU analyses used relative abundance, Hellinger transformation, and Bray-Curtis dissimilarity; incidence analyses used binary Jaccard dissimilarity. Observed richness was the number of detected VTUs in a library. The working spreadsheet's column labeled TPM equals RPM divided by contig length in kilobases; it is not conventional transcript-per-million abundance and was not used in the community analyses.

S1.3. Host-group models and sensitivity analyses

Host-group structure was tested by PERMANOVA with 9,999 permutations. Marginal models included location, standardized log10 total pre-depletion clean reads, or both, using by = 'margin'. Within-location permutation tests were performed for the unadjusted host model and, as an additional design check, for the model containing location, read depth, and host group. The latter was also fitted to the 28 libraries from nine locations with multiple host groups. Duplicate host-location profiles were averaged to give 32 profiles; the corresponding library-depth covariate was the mean total-read count within each cell. The adjusted model was repeated on these profiles with permutations restricted within location. These checks and the original unadjusted blocked-permutation models are distinguished in Table S4. The random seed was 20250715.

Feature sensitivity analyses repeated the models with weighted and binary annotation-defined VTU matrices, nucleotide-supported contigs grouped into VTUs, contigs with at least 20 mapped reads grouped into VTUs, and VTUs detected in at least two libraries. Two additional checks excluded all single-contig VTUs and repeated abundance and incidence models using the 136 multi-contig units. All 35 libraries retained at least one such unit. The location- and depth-adjusted host R^2 values were 0.2813 and 0.2792, respectively; both unblocked and location-blocked tests yielded $p = 0.0001$. P-values from the seven related location- and depth-adjusted VTU models were corrected together by the Benjamini-Hochberg method (Tables S3 and S17); all q-values were 0.0001. Single-contig lengths and library contributions are reported in Table S17. This exclusion analysis tests dependence on single-contig features; multiple contigs do not establish a complete viral genome. The checks assessed stability of the host-group association and did not validate individual viral detections. PERMDISP used the spatial-median option in vegan::betadisper and 9,999 residual permutations. Table S12 and Figure S4B report distances to these group centres. For the original Bray-Curtis representation, vegan set negative squared distances to zero when the negative-axis contribution exceeded the positive-axis contribution. We retained this analysis and added small-sample bias adjustment, followed by a check combining this adjustment with the Lingoes correction to remove negative eigenvalues. All estimates are reported in Table S12. Arithmetic-centroid distances are included descriptively for comparison; the reported permutation tests use spatial medians. The original spatial-median result was $F(6,28) = 2.450$, parametric $p = 0.0496$, and permutation $p = 0.0489$. Small-sample adjustment gave $p = 0.0440$; adding the Lingoes correction gave $p = 0.0850$. Unequal within-group dispersion therefore could not be excluded.

S1.4. Within-host spatial detections and adult-larval comparison

Within *Culex tritaeniorhynchus*, binary Jaccard dissimilarity was calculated among the 10 library-level VTU profiles. Observed site occupancy was the number of sampled locations at which a VTU was detected, counting the duplicate DL-XY libraries as one location. We repeated occupancy summaries after restricting contigs to nucleotide-supported annotations and, separately, to at least 20 mapped reads. For these checks, site-level presence was the union of detections across libraries at that site. Both possible nine-library sets with one DL-XY library were also examined. Table S16 gives the filtered counts, VTU-level site records, site-level Jaccard distances, and duplicate-site checks. These are checks of ob-

served detections, not occupancy models that estimate detection probability. For geographic distance decay, family-level relative-abundance profiles were averaged within location, including DL-XY, to give one profile for each of nine sites. Great-circle distances used longitude and latitude and a mean Earth radius of 6,371.0088 km. The two-sided Spearman Mantel test used 9,999 permutations and seed 20250714. The linear fit in Figure 9C is descriptive; inference used matrix permutations rather than treating the 36 site pairs as independent observations.

The adult-larval comparison included four adult *Culex quinquefasciatus* pools and three *Culex spp.* larval pools. Family-level relative abundance was Hellinger-transformed and ordinated by principal coordinates analysis. Stage labels were tested by PERMANOVA using complete enumeration of the 35 possible allocations of four adult labels among seven libraries. PERMDISP was evaluated separately. Because stage, species resolution, and location were confounded, the comparison was considered descriptive and exploratory.

S1.5. Application-display records and site-level host-virome comparisons

The original temperature and precipitation information was recorded from weather-application displays during sampling in June 2025, rather than measured directly at the collection sites. The reading time does not establish the represented period. The precipitation definition, unit, and accumulation interval remain unverified. The original values are retained in Tables S1 and S19 but are excluded from quantitative environmental models. The author-defined climate-zone grouping is independent of these records.

The site-level host-virome comparison includes all 15 sequenced locations after correction of two Zhaoyang field-count records previously assigned the Ludian code. This correction restored ZT-ZY without adding sequenced libraries. Field host composition used the seven taxonomically resolved categories in Table S8; unidentified field specimens were excluded. Host-count matrices were Hellinger-transformed and summarized by principal-component analysis. Library-level family relative-abundance profiles were averaged within location and Hellinger-transformed. Virome configurations were obtained by principal-coordinate analysis of Bray-Curtis dissimilarities, with an additive correction for non-Euclidean distances. The first two axes of each configuration were compared by symmetric Procrustes rotation and PROTEST with 9,999 permutations (random seed 20250714). Inputs, coordinates and results are provided in Table S19. The climate-zone analysis in S1.6 compares observed library richness and does not use the application-displayed weather values.

S1.6. Climate-zone groups, richness, and sequencing depth

The authors assigned Eco_Region labels to distinguish Yunnan plateau and southern subtropical locations, termed plateau cool/moderate and subtropical hot/wet in the figures. This is an author-defined geographical grouping, not a classification obtained by thresholding the application-displayed values recorded during sampling. The 15 sequenced locations comprised nine plateau and six subtropical sites. Observed library richness was compared between groups by a two-sided Wilcoxon rank-sum test. Cumulative VTU richness, contig count, and assigned-family count were summarized descriptively because they depend on sampling effort. The association between total reads and observed VTU richness across libraries was tested with Spearman's rank correlation. To assess unequal replication, cumulative richness was recalculated for every three-library combination within each host group; groups represented by exactly three libraries contributed one combination. A negative-binomial model of library richness included host group and log10 total reads, and adjusted means were predicted at the median library depth (40,470,473 reads). For *Culex tritaeniorhynchus*, the 120 possible three-library combinations yielded a median cumulative richness of 40.5 VTUs (range, 17-71). *Ceratopogonidae*

had 54 VTUs in its only three-library combination. The overall host term in the depth-adjusted model had likelihood-ratio p = 0.412. These analyses are reported in Table S13 and Figure S5.

Read-count rarefaction used the existing library-by-VTU count matrix and the total pre-depletion clean-read count of each of the 35 libraries. Counts were added across contigs with the same VTU label within a library; other clean reads remained in the sampling denominator. All libraries were standardized to the smallest total, 28,163,793 reads. For a VTU with c mapped reads among N clean reads, the probability of retaining at least one assigned read after sampling n reads without replacement was 1 - choose(N-c,n)/choose(N,n). Expected richness was the sum of these probabilities. This conditional calculation concerned the already retained annotations. It did not repeat assembly or annotation, reapply the original contig-retention cutoff, or estimate viruses missed during discovery. Expected richness changed by less than 0.001 VTU per library, indicating that the retained annotations were largely preserved under this count-thinning calculation. It does not establish complete virome sampling. Table S13 reports all library- and host-group-level values.

S1.7. WC-15 terminal-motif and segment-support analyses

The four WC-15 assemblies were evaluated against criteria used to characterize Jingmenvirus-related multicomponent genomes: co-detection, complementary coding functions, phylogenetic placement of the conserved nonstructural proteins, related segment-terminal sequences, and read-remapping support [40,47,48]. Coding architecture, terminal-motif similarity, phylogenetic coherence, and independent remapping were treated as the primary evidence for the candidate grouping. Segment abundance was assessed only as supporting context. Segment FASTA files were read in their supplied assembly orientation and converted to uppercase DNA notation. The first 40 nt of each assembly were searched with the regular expression ACGA[AC]{1,2}GGAT. Matches and their 1-based coordinates are listed in Table S7, and the first 35 nt are shown in Figure S2. Read-record counts, unique read sequences, and nominal depth were summarized from the segment-specific mapping outputs (Table S6; File S3). Segment RPM was calculated as valid mapped read records divided by 44,712,138 total WC-15 reads and multiplied by 10^6. RPM per assembled kilobase was also calculated to examine the effect of component length. Each abundance summary was scaled to the mean of Segments 1-3. Table S10 states the evidentiary role and limitation of each criterion.

Segment architecture and the 11,890-nt total were calculated from the assembled component FASTA files, including the 3,122-nt structural assembly listed in Table S7. Nominal read depth in Table S6 was calculated from the supplied mapping consensus, which is 3,121 nt for this component. This one-nucleotide difference is limited to the mapping consensus and does not affect the assembled total. The four RNAs were therefore treated as putative components of one candidate WC-15 Jingmenvirus-related genome. Their physical linkage was not established. Formal species assignment will require comparison against complete component-specific demarcation criteria and biological characterization [46]. Segment 4 was relatively enriched in both RPM and length-normalized summaries, consistent with observations in other Jingmenvirus-related libraries [47,48]. This pattern was not used as proof of common genome membership.

S1.8. Banna virus annotation records

Table S9 retains the original Banna virus annotation records. Its protein-search identity values are archived search outputs, not identities recalculated from the final submitted CDS annotations. The protein-query version could not be reconciled with the final VP1 CDS record; the main-text similarity comparison therefore reports the nucleotide match only.

Supplementary Figures

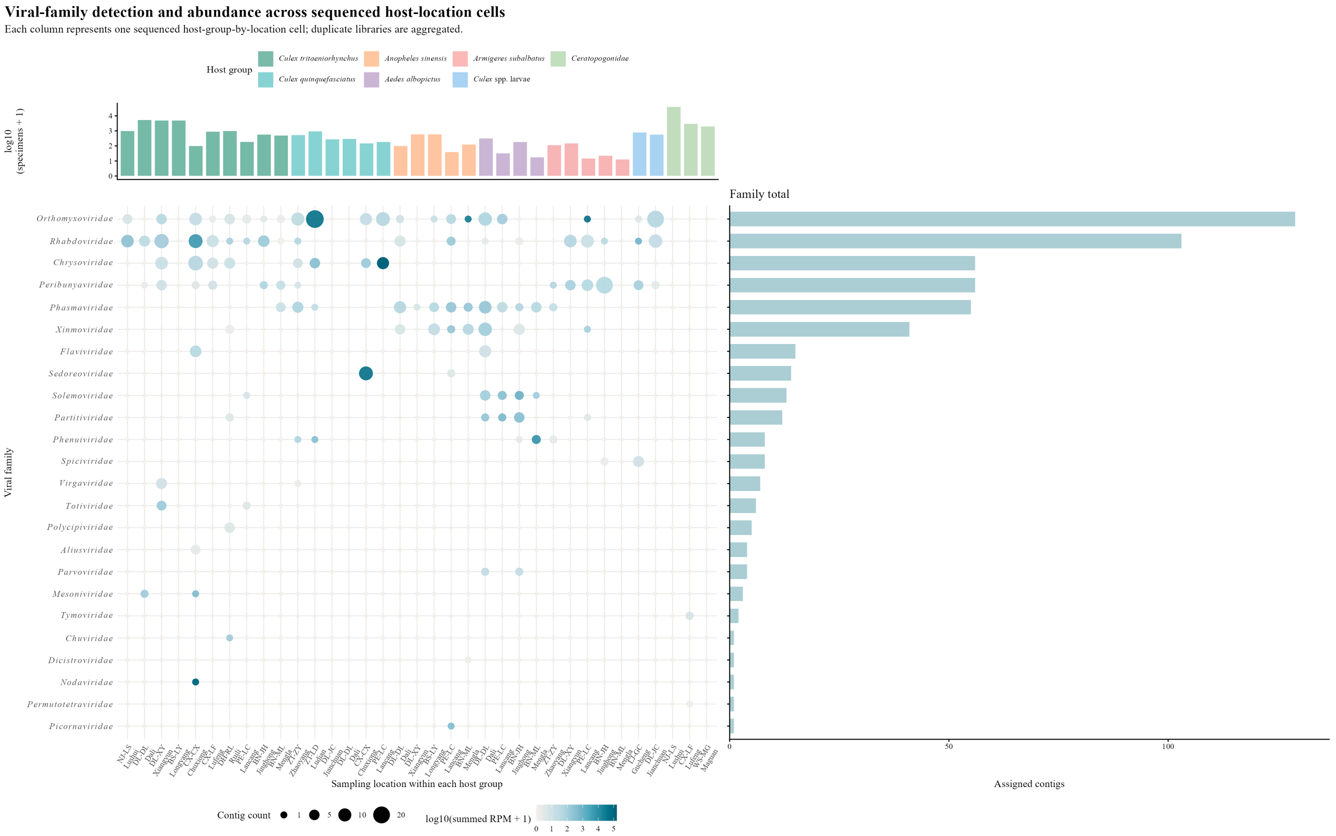

Figure S1. Viral-family profiles across 32 sequenced host-group-by-location cells. Bubble area represents the number of retained contigs assigned to each family, color represents log10(summed RPM + 1), and top bars show the corresponding field specimen counts. The three duplicate host-group-by-location library pairs are aggregated in this display. Family-unresolved contigs are not shown.

Related 5' terminal motifs in the four WC-15 candidate segments

Segment	First 35 nt of the assembled sequence	Position
NS5	G G A C T T T A A G C C A A T **A C G A A A G G A T** T G A T G G A A T A	16-25
NS3	G T T T T T T T T A A T C A A C C A T **A C G A C G G A T** T T A G G T T	20-28
VP1-like	G T T T A A T C A T C T T A T A T **A C G A A G G A T** A G A A T T T G A	18-26
Structural	G T T T T A T A G C A T T **A C G A A A G G A T** A A A C C A C T A T A A	14-23

Highlighted bases match ACGA[AC]{1,2}GGAT within the first 40 nt. Positions are 1-based.

The comparison describes assembled 5' ends; exact genomic termini have not been experimentally verified.

Figure S2. Related 5′ terminal motifs in the four WC-15 candidate components. The first 35 nt of each assembly-oriented sequence are shown; colored boxes mark motifs matching ACGA[AC]{1,2}GGAT within the first 40 nt. Positions are 1-based. Read support is reported in Table S6, motif coordinates are listed in Table S7, and the complete grouping rationale is summarized in Table S10.

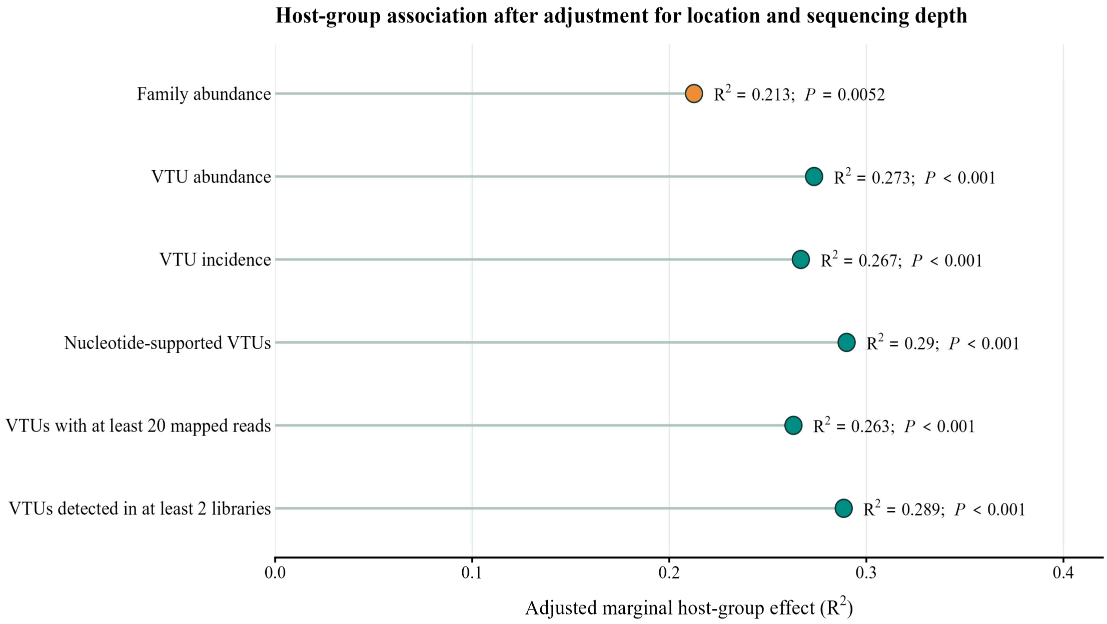

Figure S3. Host-group-associated virome structure after adjustment for sampling location and sequencing depth. Points show the marginal host-group R^2 from models that included both covariates. Estimates are shown for family abundance, weighted and binary annotation-defined VTUs, nucleotide-supported VTUs, detections with at least 20 reads, and VTUs found in at least two libraries. Exact model results are listed in Table S3.

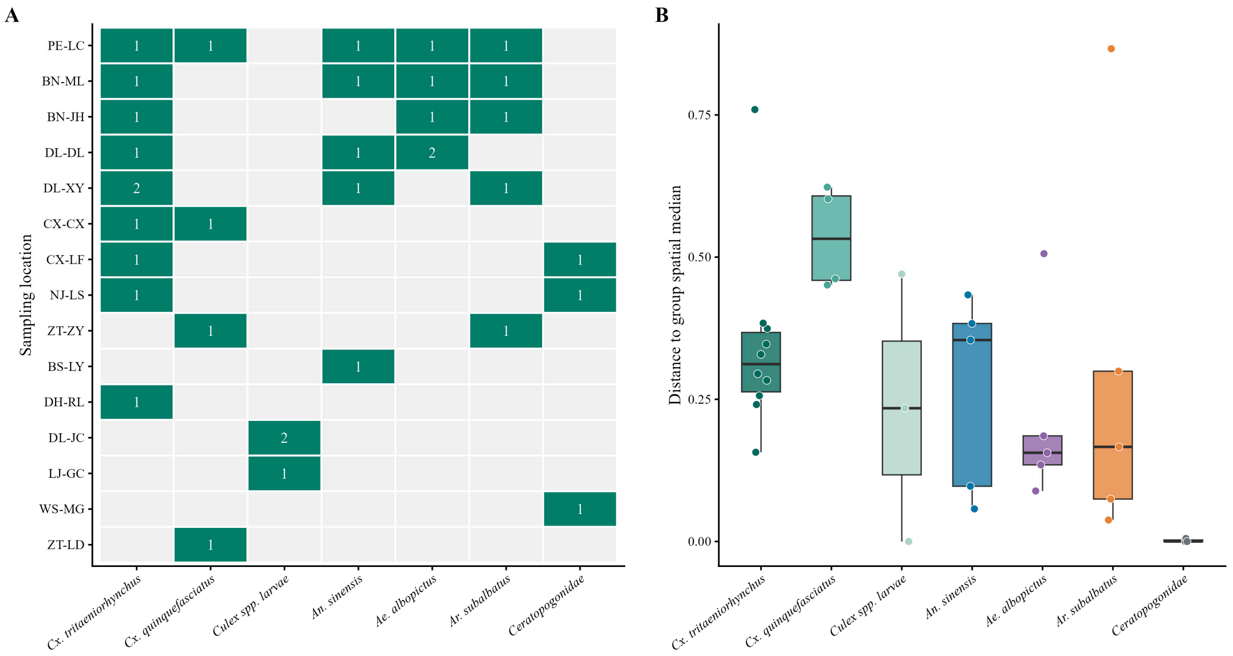

Figure S4. Sampling overlap and within-group multivariate dispersion. (A) Number of sequenced libraries for each host group at all 15 locations. Nine locations contained at least two sequenced host groups. (B) Library-level distances to the host-group spatial median from the original family-level Bray-Curtis PERMDISP analysis. Points represent individual libraries; boxes show the median and interquartile range, and whiskers extend to observations within 1.5 times that range. Larger distances indicate greater within-group heterogeneity. Tables S11 and S12 provide exact values, arithmetic-centroid distances, and the small-sample and Lingoes sensitivity checks.

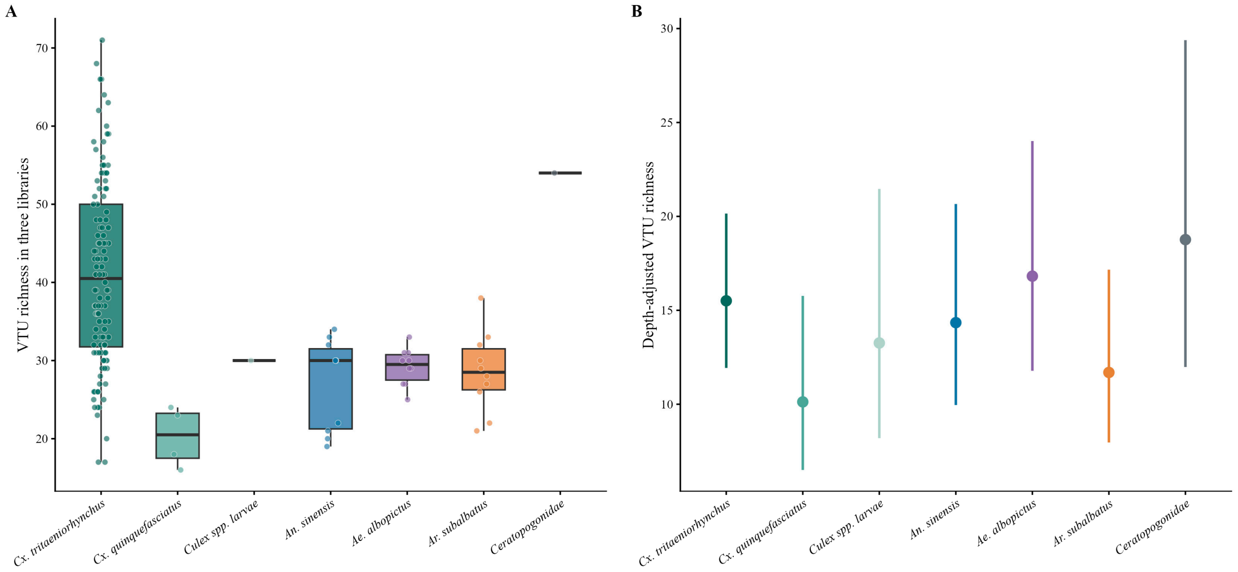

Figure S5. Sensitivity of annotation-defined VTU richness to sampling effort and sequencing depth. (A) Cumulative richness from every three-library combination within each host group; points show combinations and boxes summarize groups with multiple combinations. (B) Negative-binomial adjusted mean library richness predicted at the median sequencing depth, with 95% confidence intervals. The overall host-group term was not significant (likelihood-ratio p = 0.412). Exact values are listed in Table S13.

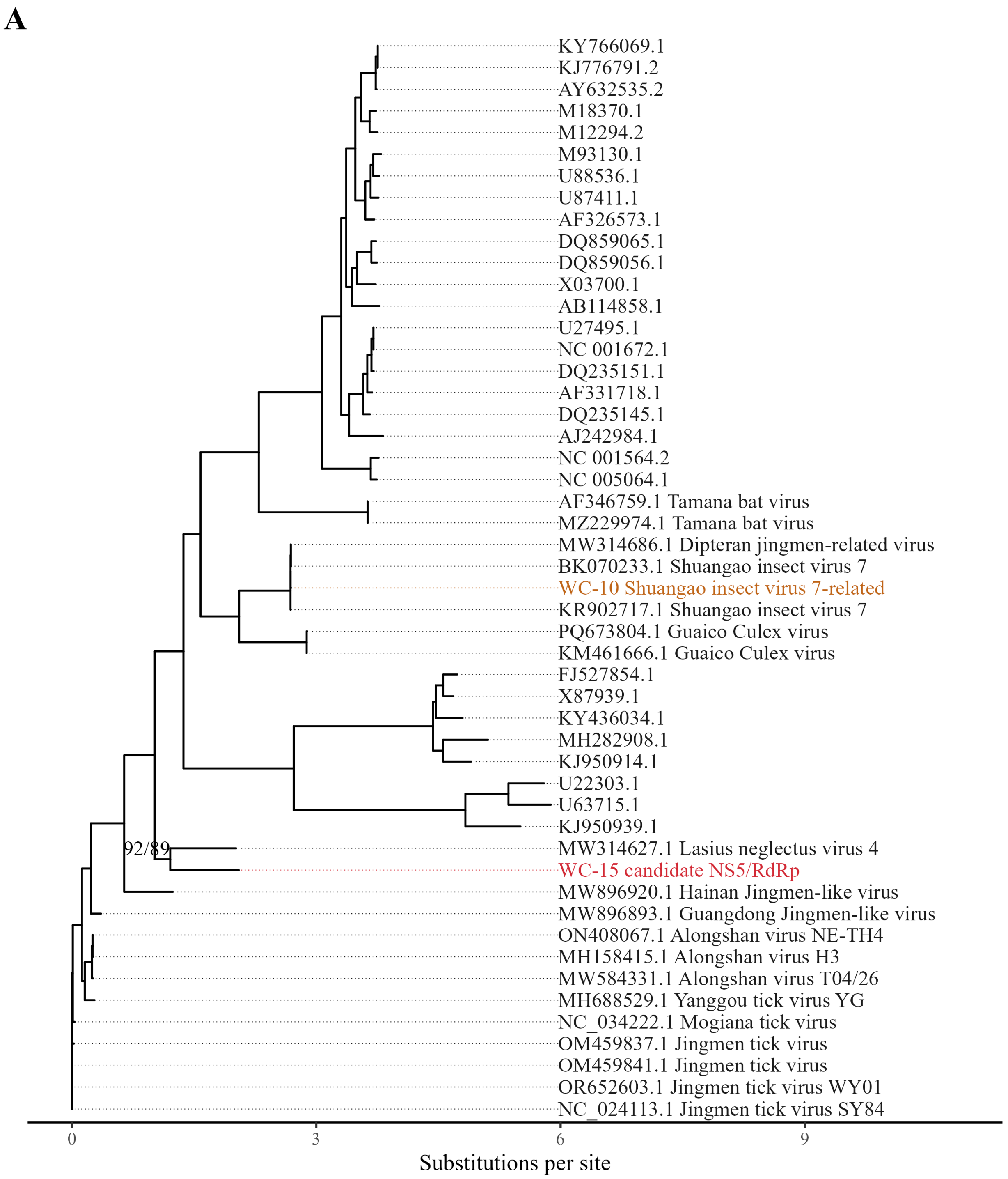

Figure S6A. Full maximum-likelihood NS5/RdRp tree underlying Figure 6A. All retained tips are shown; WC-15 is highlighted in red. Focal labels give SH-aLRT/UFBoot support. Branch lengths are substitutions per site. Display orientation follows the saved unrooted tree, without additional rerooting. Source trees, alignments, and reports are provided in File S2; panel-specific records are in Table S14c.

B

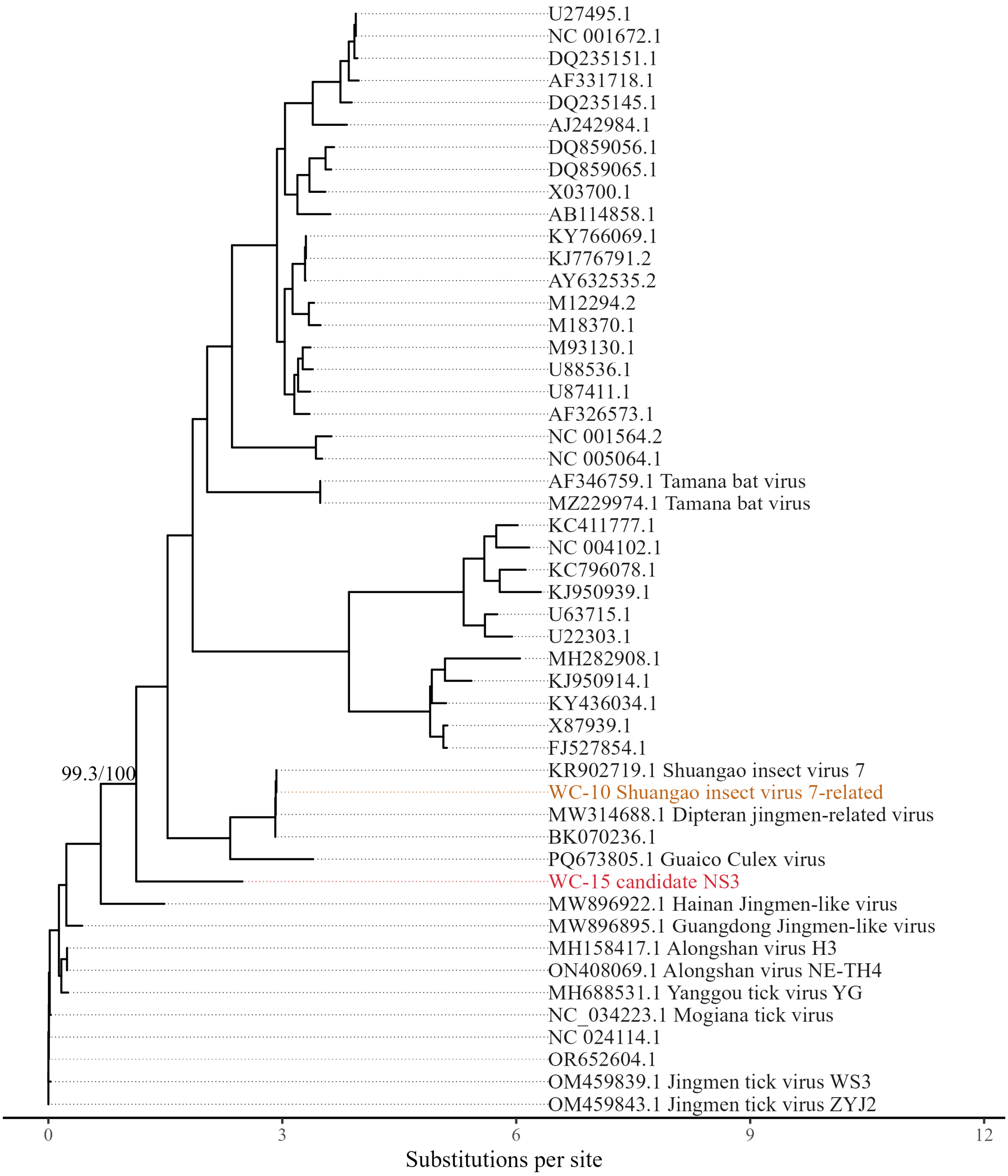

Figure S6B. Full maximum-likelihood NS3 tree underlying Figure 6B. All retained tips are shown; WC-15 is highlighted in red. Focal labels give SH-aLRT/UFBoot support. Branch lengths are substitutions per site. Display orientation follows the saved unrooted tree, without additional rerooting. Source trees, alignments, and reports are provided in File S2; panel-specific records are in Table S14c.
